# β-hydroxybutyrate recapitulates the beneficial effects of ketogenic metabolic therapy in polycystic kidney disease

**DOI:** 10.1016/j.isci.2024.110773

**Published:** 2024-08-20

**Authors:** Jacob A. Torres, Nickolas Holznecht, David A. Asplund, Bradley C. Kroes, Tselmeg Amarlkhagva, Matthias M. Haeffner, Elizabeth H. Sharpe, Stella Koestner, Sebastian Strubl, Margaret F. Schimmel, Samantha Kruger, Shagun Agrawal, Brina A. Aceves, Muthusamy Thangaraju, Thomas Weimbs

**Affiliations:** 1 Department of Molecular, Cellular, Developmental Biology, University of California Santa Barbara, Santa Barbara, CA, USA; 2 Department of Biochemistry and Molecular Biology, University of Augusta, Augusta, GA, USA

**Keywords:** Therapy, Pathophysiology, Diet

## Abstract

Autosomal-dominant polycystic kidney disease (ADPKD) is a common monogenic disease characterized by the formation of fluid-filled renal cysts, loss of mitochondrial function, decreased fatty acid oxidation, increased glycolysis, and likely renal failure. We previously demonstrated that inducing a state of ketosis ameliorates or reverses PKD progression in multiple animal models. In this study, we compare time-restricted feeding and 48-h periodic fasting regimens in both juvenile and adult Cy/+ rats. Both fasting regimens potently prevent juvenile disease progression and partially reverse PKD in adults. To explore the mechanism of fasting, we administered β-hydroxybutyrate (BHB) to Cy/+ rats and orthologous mouse models of PKD (*Pkd1*^*RC/RC*^, *Pkd1-Ksp:Cre*). BHB recapitulated the effects of fasting in these models independent of stereoisomer, suggesting the effects of BHB are largely due to its signaling functions. These findings implicate the use of ketogenic metabolic therapy and BHB supplementation as potential disease modifiers of PKD and point toward underlying mechanisms.

## Highlights

• Polycystic kidney disease leads to increased glycolysis and metabolic disorder
• Ketogenic therapy may alter disease outcomes by addressing the central metabolic issue
• Both fasting and time-restricted feeding slow or reverse disease progression in rodents
• We show that β-hydroxybutyrate alone recapitulates the benefits of fasting

## Graphical abstract

**Figure:**
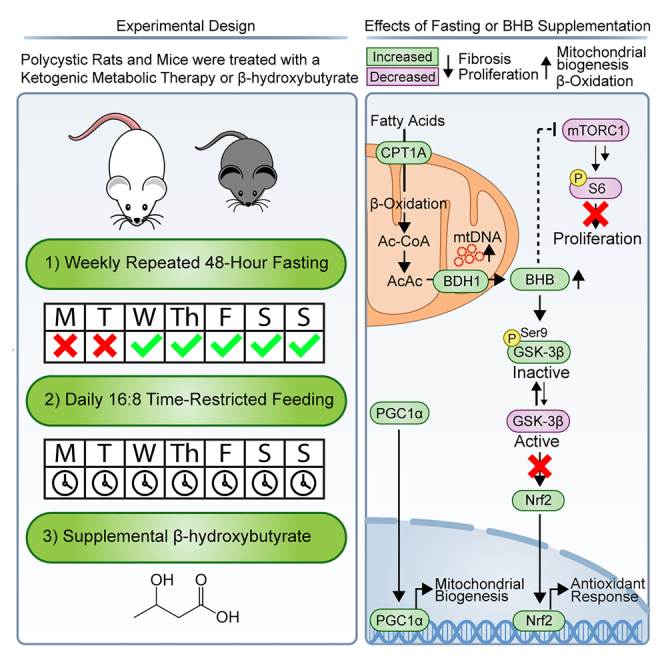

## Introduction

Autosomal dominant polycystic kidney disease (ADPKD) results from mutations in the *PKD1* or *PKD2* genes and affects between 1:400–1:1000 individuals worldwide, making it the leading, life-threatening monogenic illness.^1^ The need for dialysis and kidney transplantation is the norm for most individuals with ADPKD.^1^ Tolvaptan, a vasopressin-V2 receptor antagonist, is currently the only approved pharmacological therapy for ADPKD. However, Tolvaptan does not stop disease progression, has significant adverse effects and toxicities, and its high cost limits availability for patients worldwide,^2^^,^^3^ making effective, safe, and economical therapies for ADPKD urgently required.

Recently, ADPKD has gained recognition as a metabolic disorder^4^^,^^5^^,^^6^^,^^7^^,^^8^ wherein cyst cells become highly dependent on glucose and glycolysis to meet their energy requirements. The increased glycolytic activity accompanies impaired mitochondrial structure and function, leading to defective fatty acid oxidation, akin to the cancer cell Warburg effect.^9^^,^^10^^,^^11^^,^^12^^,^^13^ To address this, pharmacological inhibition of glycolysis^14^ and approaches to improve mitochondrial function have shown efficacy in preclinical studies in altering disease progression.^15^^,^^16^^,^^17^^,^^18^^,^^19^^,^^20^ The peroxisome proliferator-activated receptor (PPAR) agonists rosiglitazone^15^ and fenofibrate,^16^ the Nrf2 activator sulforaphane,^17^ and the AMP-activated protein kinase (AMPK) activator metformin^18^^,^^19^^,^^20^ slow disease progression in multiple animal models. The Nrf2 activator bardoxolone completed a phase 2 clinical trial for ADPKD, but the phase 3 trial was terminated early by the manufacturer due to insufficient efficacy at preventing end-stage kidney disease in another phase 3 trial using the same drug,^21^ leaving open the question whether Nrf2 activation is an effective strategy in ADPKD. The optimism for utilizing targeted metabolic reprogramming is high and suggests that more conservative and economical options such as ketogenic metabolic therapy (KMT) may be an effective strategy for managing ADPKD.

KMT includes caloric restriction, time-restricted feeding (TRF), extended fasting, and a ketogenic diet, all of which ameliorate disease progression in multiple animal models of PKD.^4^^,^^5^^,^^6^ We previously showed that different KMT interventions are highly effective in multiple PKD animal models, including a ketogenic diet that almost completely prevents disease progression in young rats with PKD and even partially reverses established renal cystic disease in adult rats.^5^ Furthermore, supplementation with the primary ketone produced during ketosis, β-hydroxybutyrate (BHB), mimicked the beneficial effects of nutritional ketosis.^5^

These preclinical studies have led to clinical translation using KMT in ADPKD with promising results. Our retrospective case series study of 131 individuals with ADPKD who self-reported having undertaken KMT (mostly ketogenic diets) for an average duration of 6 months suggested beneficial effects on overweight/obesity, BMI reduction, improvement in pain, and other ADPKD-related symptoms, alongside significant self-reported improvement of both hypertension and eGFR.^22^ Similarly, a 3-month, dietitian-directed KMT intervention program specifically created for ADPKD combines a plant-focused ketogenic lifestyle approach, with a reduction of renal stressors and a novel medical food containing exogenous BHB and alkaline citrate.^23^ Results from a pilot study^23^ using this approach and ongoing experience with over 150 individuals who have completed the program suggest that the intervention improves hypertension and kidney pain and may beneficially affect renal function. Additionally, a randomized controlled, 3-month pilot trial (KETO-ADPKD) with 66 ADPKD patients comparing periodic water fasting vs. a ketogenic diet vs. no dietary change confirmed safety and feasibility, and found a significant reduction in body fat, and a significant improvement in eGFR (based both on creatinine and cystatin C).^24^ Post-hoc subgroup analysis of individuals with consistent ketone levels also showed a significant reduction in total kidney volume in the ketogenic diet group.^25^ The effect size found with KMT in the KETO-ADPKD trial far surpasses any previously reported effect sizes with other treatments since no other trial has ever shown improvement in renal function or a decrease in total kidney volume. These data all suggest a disease-modifying benefit of using KMT for PKD therapy but additional clinical trials will be required in order to ascertain long-term efficacy. Several ongoing or upcoming controlled clinical trials will evaluate long-term outcomes of KMT on metabolic and renal health in ADPKD.

While ketogenic diet approaches have already started to receive a great deal of interest in ADPKD disease management, the relatively restrictive nature of such a diet may limit widespread adoption. In contrast to restrictive dietary interventions, alternate methods of achieving the goals of KMT, such as TRF or BHB supplementation, are easily accessible, require no change in food choices, and may lead to increased adherence. Although extended day fasts may be contraindicated for some individuals, TRF appears safe for nearly everyone when appropriately implemented. TRF also has benefits compared to ketogenic dieting and extended fasts, as it can easily integrate into a daily routine. Its flexibility also allows for social activities that involve eating.

In this study, we expand our previous findings with juvenile PKD rats treated with TRF by performing additional TRF experiments in adult rats with established PKD.^5^ We also observed that a single 48-h fast dramatically reduced the size of cysts in adult rats^5^ and aimed to elucidate the potentially beneficial effect of repeated weekly 48-h fasts (periodic fasting, PF) by directly comparing the effects of PF to TRF in both juveniles and adults. Finally, we previously demonstrated that the beneficial effects of KMT were recapitulated with exogenous BHB in juvenile rats and are now testing the effect of BHB in adult rats and orthologous *Pkd1*^*RC/RC*^*,* and neonatal *Pkd1*^*fl/fl*^*-Ksp:Cre* mouse models to determine if BHB alone is sufficient to recapitulate the effects of fasting.

## Results

### Periodic fasting and time-restricted feeding ameliorate polycystic kidney disease progression in juvenile Cy/+ rats

Our previous study on KMT interventions with the Han:SPRD (Cy/+) rat model utilized 3-week-old rats placed on a 16:8 TRF regimen wherein food was provided *ad libitum* for 8 h each day and then restricted for the remaining 16 h. This intervention led to the prevention of cyst growth and reduced markers of cystic disease.^5^ We also observed that a single 48-h fast in adult rats rapidly decreased cyst size and was accompanied by an increase in cystic epithelial apoptosis and denudation. These surprising findings raised the question as to whether the effect of repeated fasting had similar or greater efficacy to that of TRF and whether BHB might mediate these effects.

For our primary analysis of the effects of PF and TRF we utilized male Cy/+ rats. Male Cy/+ rats experience a more severe form of PKD compared to females.^26^ We have included females in our study for the sake of completeness but will primarily focus on the effects of dietary interventions in males.

**Figure 1:**
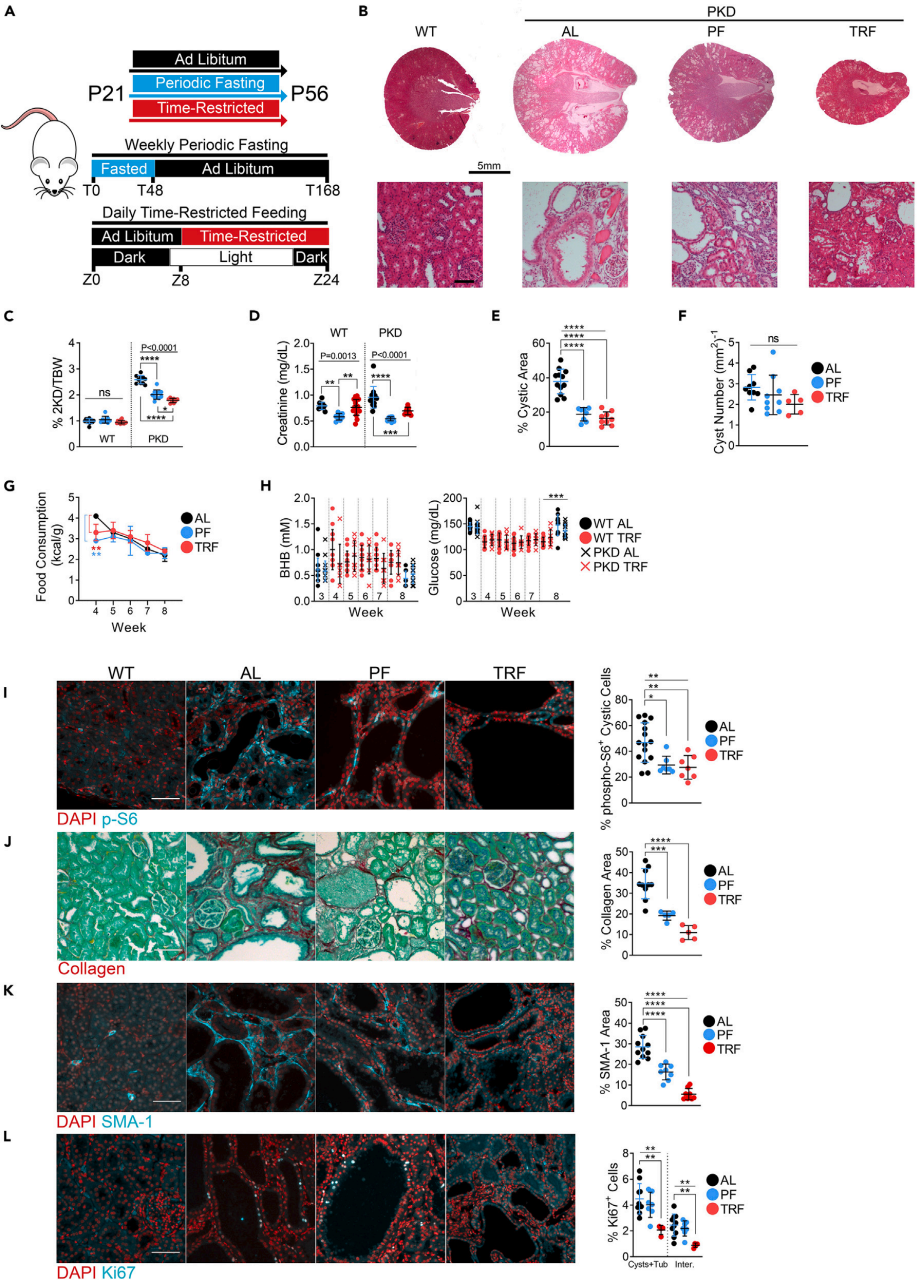
Periodic fasting and time-restricted feeding ameliorate polycystic kidney disease progression in juvenile Cy/+ Rats (A) Schematic of feeding and experimental designs for juvenile rat experiments. (B) Hematoxylin and Eosin stained kidneys from male wild-type and male polycystic *ad libitum*, PF and TRF rats. Scale = 5mm (Top); 100μm (Bottom). (C) 2-kidney over body weight of *ad libitum*, PF and TRF male wild-type and polycystic rats. (D) Serum creatinine of *ad libitum*, PF and TRF male wild-type and polycystic rats. (E) Cystic area of *ad libitum*, PF and TRF male wild-type and polycystic rats. (F) Cyst number per mm^2^ of kidney from *ad libitum*, PF and TRF male wild-type and polycystic rats. (G) Weekly food consumption of *ad libitum*, PF and TRF male rats. (H) Blood BHB and blood glucose of *ad libitum*, PF and TRF male wild-type and polycystic rats. (I) Immunofluorescence of phospho-S6^235/236^ and quantification of cytoplasmic phospho-S6^235/236^ within cystic epithelia in male wild-type *ad libitum*, and male polycystic *ad libitum*, PF and TRF rats. Scale = 50μm. (J) Sirius Red and Fast Green stain for collagen (red) and quantification from male wild-type *ad libitum* and male polycystic *ad libitum*, PF and TRF rats. Scale = 70μm. (K) Immunofluorescence of Smooth Muscle Actin (SMA-1) and quantification from male wild-type *ad libitum* and male polycystic *ad libitum*, PF and TRF rats. Scale = 50μm. (L) Immunofluorescence of Ki67 and quantification from male wild-type *ad libitum* and male polycystic *ad libitum*, PF and TRF rats. Scale = 50μm. (See also Figures S1 and S2. One-way ANOVA followed by ad hoc Tukey’s test was used for multiple comparisons. A paired t-test was used for single groups. Mean and standard deviations are shown. ∗ = *p* < 0.05, ∗∗∗ = *p* < 0.01, ∗∗ = *p* < 0.001, ∗∗∗∗ = *p* < 0.0001).

To directly compare the results of our previous findings with TRF to PF in PKD, we utilized 3-week-old (P21) Cy/+ rats and restricted food access for 48 h, followed by *ad libitum* access for five days, and then repeated the 48-h fast four additional times. TRF-treated rats had *ad libitum* food access for 8 h daily and were food-restricted for the remaining 16 h for five weeks (Figure 1A).

Compared to *ad libitum* (AL) fed rats, both PF and TRF regimens significantly slowed renal cystic progression (Figure 1B), led to reduced 2-kidney to bodyweight ratios (Figures 1C, S1A, and S2A), serum creatinine (Figures 1D and S2B), cystic area (Figures 1E and S2C) and a non-significant reduction in cyst numbers (Figures 1F and S2D).

The beneficial effects of PF and TRF do not appear to be due to caloric restriction since PF and TRF-treated rats consumed comparable calories to AL rats after the first week of diet modification (Figure 1G). PF-treated rats ended the study with less body mass than TRF and AL rats (Figures S1B–S1D and S2A), whereas TRF and AL rats followed similar growth curves (Figures S1D and S1E). Additionally, PF-treated rats did appear to consume slightly more water than AL rats during the 5-week period (Figure S1F).TRF-treated rats showed on average a non-significant increase in blood BHB and decreased glucose levels during treatment compared to baseline (Figures 1H and S1G).

Our lab and others have shown that mTOR is a crucial driver of cyst growth in PKD.^27^^,^^28^^,^^29^^,^^30^ Our previous study using TRF found that ketogenic interventions decrease phospho-S6^235/236^ (pS6), a marker of S6 kinase (S6K) activity, a downstream target of mTOR complex 1 (mTORC1), within cystic epithelia.^5^ As shown in Figure 1I, PF and TRF strongly reduce pS6 positivity within cystic epithelia, suggesting a potent inhibitory effect on mTOR activity (Figure 1I).

To investigate the effects on renal fibrosis, we examined collagen deposition by Sirius Red staining and found it strongly reduced in PF and TRF-treated animals compared to AL controls (Figure 1J). Collagen-producing SMA-1 positive myofibroblasts are abundant in cystic kidneys in AL-fed animals but significantly diminished after TRF or PF treatment, with a stronger effect measured in TRF rats (Figure 1K).

The cell cycle marker Ki67 was also significantly decreased with TRF in both cystic/tubule and interstitial cells but not PF (Figure 1L), indicating decreased proliferation specific to TRF.

### Periodic fasting and time-restricted feeding ameliorate and reverse polycystic kidney disease progression in adult Cy/+ rats

**Figure 2:**
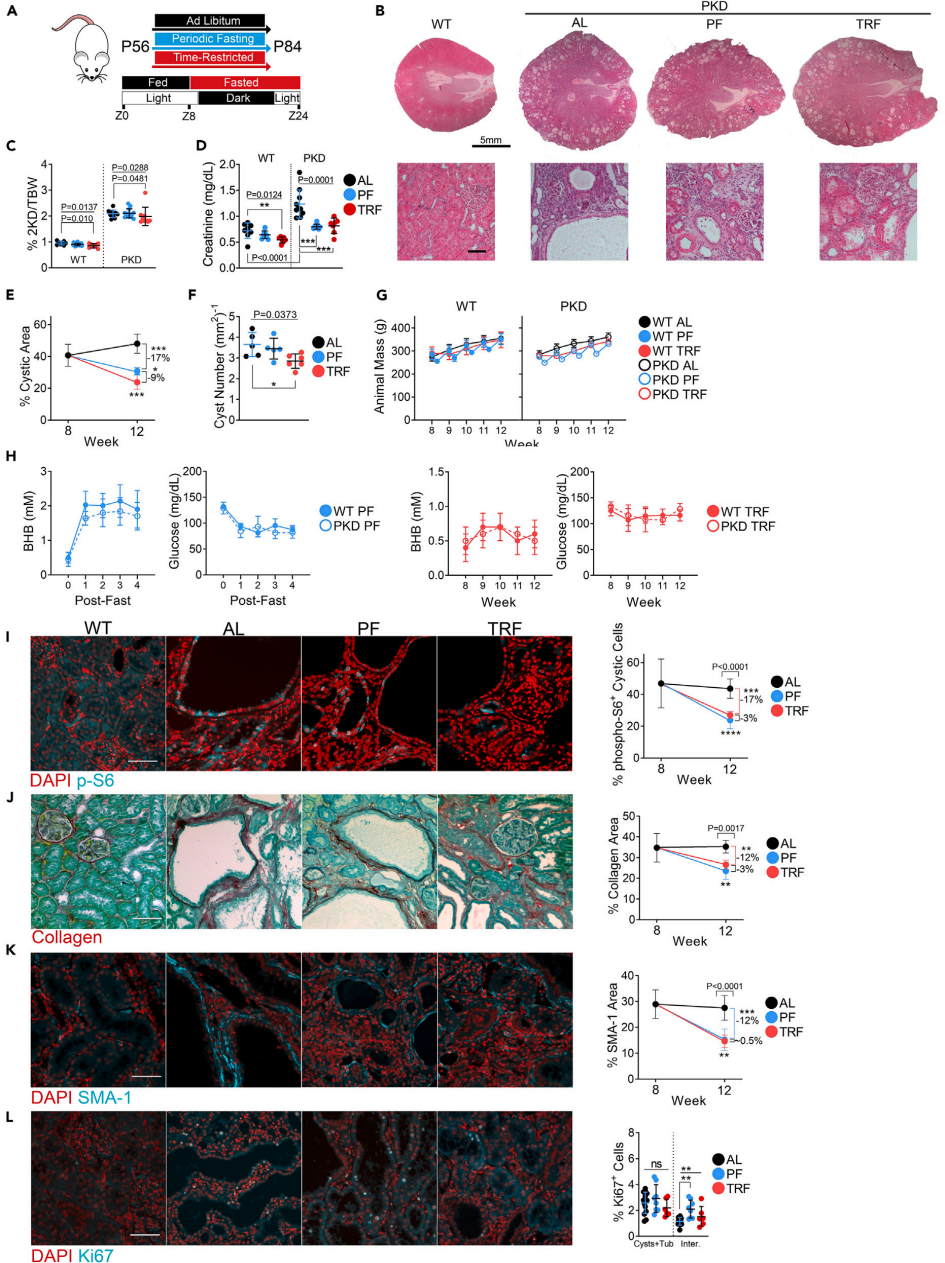
Periodic fasting and time-restricted feeding ameliorate and reverse polycystic kidney disease progression in adult Cy/+ Rats (A) Schematic of PF and TRF feeding experimental timeline and time-restricted feeding schedule for adult Cy/+ rats. (B) Hematoxylin and Eosin stained of 12-week-old male wild-type *ad libitum* and male polycystic *ad libitum*, PF and TRF rats. Scale = 5mm (Top); 100μm (Bottom). (C) 2-kidney over body weight of *ad libitum*, PF and TRF male wild-type and polycystic rats. (D) Serum creatinine of *ad libitum*, PF and TRF male wild-type and polycystic rats. (E) Change in the cystic area between 8-week-old *ad libitum* and 12-week-old *ad libitum*, PF and TRF male polycystic rats. (F) Cyst number per mm^2^ of kidney from *ad libitum*, PF and TRF male polycystic rats. (G) Weekly average animal masses of *ad libitum*, PF and TRF male wild-type and polycystic rats. (H) Weekly blood BHB and blood glucose averages from male wild-type and polycystic rats. (Left) Blood measurements were taken before fasting (time 0) and at the end of each 2-day fast for periodic fasted rats. (Right) The weekly blood values for time-restricted rats collected at the end of the time-restricted fasting period. (I) Immunofluorescence of phospho-S6^235/236^ and the change in cytoplasmic phospho-S6^235/236^ signals within cystic epithelia between 8-week-old *ad libitum* and 12-week-old *ad libitum*, PF and TRF male polycystic rats. Scale = 50μm. (J) Sirius Red and Fast Green stain for collagen (red) and the change in collagen deposition between 8-week-old *ad libitum* and 12-week-old *ad libitum*, PF and TRF male polycystic rats. (K) Immunofluorescence of Smooth Muscle Actin (SMA-1) and the change in SMA-1 signal between 8-week-old *ad libitum* and 12-week-old *ad libitum*, PF and TRF male polycystic rats. Scale = 50μm Scale = 70μm. (L) Immunofluorescence of Ki67 and the quantification of Ki67 signal in cysts/tubules and interstitial cells from *ad libitum*, PF and TRF adult male polycystic rats. Scale = 50μm. (See also Figures S3–S6. One-way ANOVA followed by ad hoc Tukey’s test was used for multiple comparisons. Mean and standard deviations are shown. ∗ = *p* < 0.05, ∗∗ = *p* < 0.01, ∗∗∗ = *p* < 0.001, ψ = *p* < 0.0001).

Since both PF and TRF treatments inhibited the development of PKD during the phase of rapid cyst growth in juvenile Cy/+ rats, we next asked whether PF and TRF treatments may also affect established renal cystic disease in older animals. To test this, we treated adult 8-week-old rats for four weeks with either PF or TRF and compared them to control AL-fed animals (Figure 2A).

Similar to the juvenile model, both PF and TRF treatments altered the progression of kidney disease in adult animals (Figure 2B). The 2-kidney to bodyweight ratio decreased with TRF but not PF treatment (Figures 2C, S3A, S4A, and S5A). Serum creatinine improved in PF and TRF compared to AL (Figure 2D). The percent cystic area increased in AL controls between 8 and 12 weeks during the treatment period, indicating ongoing cyst expansion. In contrast, PF and TRF led to significant decreases in the cystic burden, suggesting a partial reversal of cystic disease (Figures 2E, S3B, S4B, and S4C). However, only male TRF-treated (Figure 2F) and female PF-treated rats (Figure S4D) showed a reduced total cyst number. AL, PF, and TRF regimens led to comparable calorie consumption over the treatment period (Figure S3C), indicating that the beneficial effect is not solely due to caloric restriction but rather nutrient timing. Apart from the first week, growth rates and animal masses were similar between the AL, PF, and TRF groups (Figures 2G, S3D, and S3E).

We measured BHB and blood glucose levels weekly and observed an increase in BHB and a depression in blood glucose in both PF and TRF rats compared to baseline measurements. Due to the nature of the restriction, PF-treated rats exhibited more strongly increased BHB and decreased blood glucose post-fast compared to TRF-treated rats (Figures 2H, S3F, and S3G).

IGF-1 contributes to the progression of PKD,^31^ and the blunting of IGF-1 is a known effect of fasting.^32^ We analyzed serum IGF-1 levels and found that TRF blunted circulating IGF-1 (Figure S5B).

We analyzed measures of PKD and found that both PF and TRF treatments decreased pS6 expression in cystic epithelia compared to 12-week AL-fed rats (Figures 2I and S6A) and similarly decreased collagen deposition (Figures 2J and S6B) and SMA-1 positive myofibroblasts (Figures 2K and S6C). Ki67 expression did not change in cystic/tubule cells but slightly increased in interstitial cells of PF-treated rats (Figure 2L).

### Periodic fasting and time-restricted feeding increases Nrf2 expression, GSK-3β Phosphorylation and proteins for fatty acid oxidation

**Figure 3:**
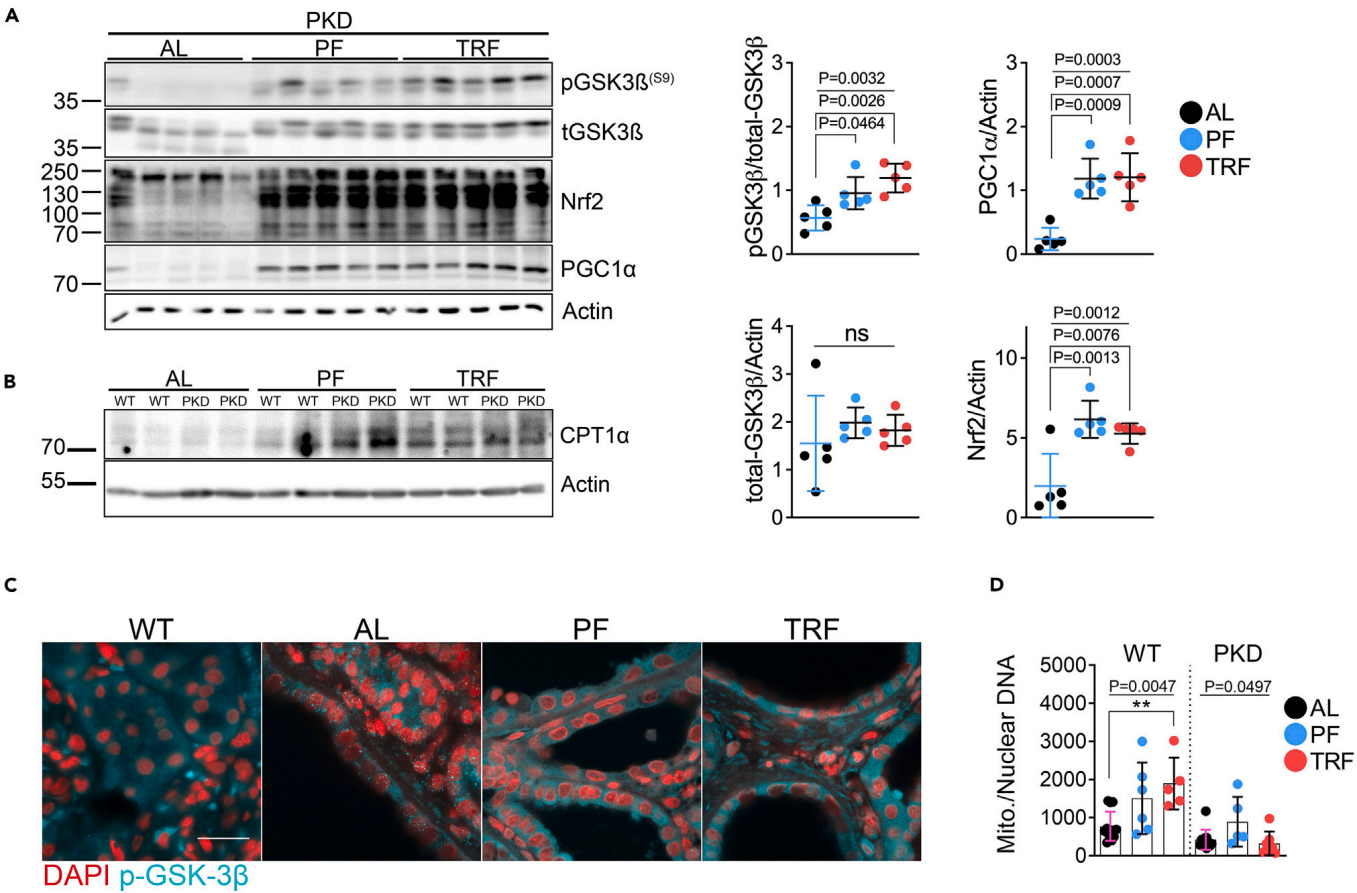
Periodic fasting and time-restricted feeding increases Nrf2 expression, GSK-3ß phosphorylation and proteins for fatty acid oxidation (A) Western blot and quantification of total and phosphorylated GSK-3β^S9^, Nrf2, and PGC1α from 12-week-old *ad libitum*, PF and TRF male polycystic rat kidneys. (B) Western blot of CPT1α from 12-week-old *ad libitum*, PF and TRF male polycystic rat kidneys. (C) Immunofluorescence of phosphorylated-GSK-3β^S9^ in male wild-type *ad libitum* and male polycystic *ad libitum*, PF and TRF rats. Scale = 20μm. (D) Quantification of mitochondrial and nuclear DNA from 12-week-old *ad libitum*, PF and TRF male wild-type and cystic rat kidneys. (See also Figures S3–S6. One-way ANOVA followed by ad hoc Tukey’s test was used for multiple comparisons. Mean and standard deviations are shown. ∗ = *p* < 0.05, ∗∗ = *p* < 0.01).

We next performed western blot analyses to determine the effects of feeding on PKD-associated signaling pathways. We detected substantial increases in phospho-GSK-3β^S9^ (pGSK-3β), a known inhibitor of mTORC1 signaling,^33^ the redox-sensitive transcription factor Nuclear factor erythroid 2-related factor 2 (Nrf2), and the mitochondrial biogenic protein PGC1α (Figures 3A and S6D) in PF and TRF-treated rats relative to AL controls. We also detected increased expression of the mitochondrial fatty acid transporter carnitine palmitoyl transferase-1 alpha (CPT1α), a marker of improved fatty acid oxidation (Figure 3B).

Interestingly, we observed increased pGSK-3β in PF and TRF cystic epithelia alongside altered cellular localization compared to AL controls (Figure 3C). The changes in pGSK-3β, Nrf2, PGC1α, and CPT1α expression suggest a potential improvement in mitochondrial function. Therefore, we measured the total mitochondrial DNA content in 12-week rats. PF and TRF treatments increased mitochondrial DNA copy numbers in wild-type rats, whereas only PF increased mitochondrial DNA copy numbers in cystic rats (Figure 3D).

### BHB alone reverses signs of polycystic kidney disease in adult Cy/+ rats

**Figure 4:**
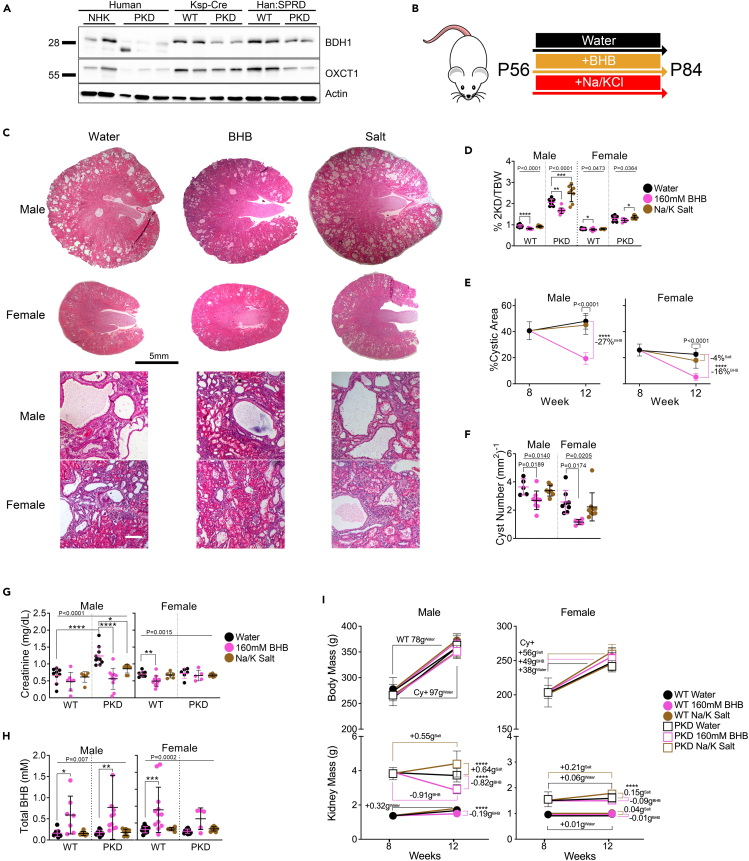
BHB alone reverses signs of polycystic kidney disease in adult Cy/+ Rats (A) Western blot of β-hydroxybutyrate dehydrogenase 1 (BDH1) and 3-oxoacid CoA-transferase (OXCT1) from human, *Pkd1*-Ksp:Cre and Han:SPRD (Cy/+) rat wild-type and polycystic kidneys. NHK = Normal Human Kidney. (B) Treatment scheme for adult BHB experiments. (C) Hematoxylin and eosin stained kidneys from 12-week-old polycystic rats supplemented with water, 160mM BHB, or sodium/potassium salts in drinking water. Scale bar = 5mm (Top) and 50μm (Bottom). (D) 2-kidney over body weight of water, 160mM BHB, and salt-supplemented wild-type and polycystic rats. (E) Change in the cystic area between 8-week-old water and 12-week-old water, 160mM BHB, and salt-supplemented male and female polycystic rats. (F) Quantification of the number of cysts per mm^2^ from whole kidney sections of water, 160mM BHB, and salt-supplemented male and female polycystic rats. (G) Serum creatinine from water, 160mM BHB, and salt-supplemented male and female wild-type and polycystic rats. (H) Serum total-BHB (L and D-BHB combined) values from water, 160mM BHB, and salt-supplemented male and female wild-type and polycystic rats. (I) Changes in kidney and body masses between 8-week-old water and 12-week-old water, 160mM BHB, and salt-supplemented male and female wild-type and polycystic rats. (See also Figure S7. One-way ANOVA followed by ad hoc Tukey’s test was used for multiple comparisons. Mean and standard deviations are shown. ∗ = *p* < 0.05, ∗∗ = *p* < 0.01, ∗∗∗ = *p* < 0.001, ∗∗∗∗ = *p* < 0.0001).

We analyzed PKD and normal human, mouse, and rat kidneys for crucial enzymes needed for the metabolism of BHB and found that the enzymes β-hydroxybutyrate dehydrogenase 1 (BDH1) and 3-oxoacid CoA-transferase 1 (OXCT1) were decreased in human and rodent PKD kidneys (Figure 4A), indicating possible impairment of BHB production and utilization by cystic kidneys.

We previously showed that supplementing juvenile rats with exogenous BHB in drinking water effectively prevented cystic disease progression.^5^ To investigate whether BHB supplementation may also have beneficial effects on fully established renal cystic disease, we utilized 8-week-old Cy/+ rats and supplemented them with racemic BHB (as sodium/potassium salt) in water *ad libitum* for four weeks and compared this to sodium/potassium chloride-supplemented controls (Na/K salt) (Figure 4B). BHB supplementation significantly improved PKD in both male and female rats while Na/K salt supplementation exacerbated it (Figure 4C). BHB supplementation also reduced the 2-kidney to body weight in both PKD and wild-type rats (Figure 4D). The effect on 2-kidney to body weight in wild-type rats is attributable to both an increase in body mass and a decrease in kidney mass specific to BHB supplementation (Figure S7A). Additionally, Na/K salt treatment increased heart hypertrophy in male cystic rats, but was prevented by BHB supplementation (Figure S7A). Compared to the start of treatment at eight weeks, BHB caused a partial reversal of cystic disease by the endpoint at 12 weeks, as evidenced by reduced cystic area (Figures 4E and S7B) cyst size, (Figure S7C) and cyst numbers (Figures 4F and S7D). Serum creatinine improved with BHB in male PKD rats, indicating the preservation of renal function (Figure 4G).

BHB supplementation significantly increased total-serum BHB levels (Figure 4H), when combining D and L-BHB isomers (Figure S7E). BHB supplementation did not decrease serum glucose in PKD animals, suggesting that its beneficial effect is not due to limiting glucose availability (Figure S7F). BHB supplementation reduced kidney size in PKD and male wild-type kidneys while Na/K increased kidney size. With neither BHB or Na/K significantly altering animal growth (Figure 4I).

We measured daily food and water intake and found that BHB supplementation slightly increased water but not food intake (Figure S7G).

**Figure 5:**
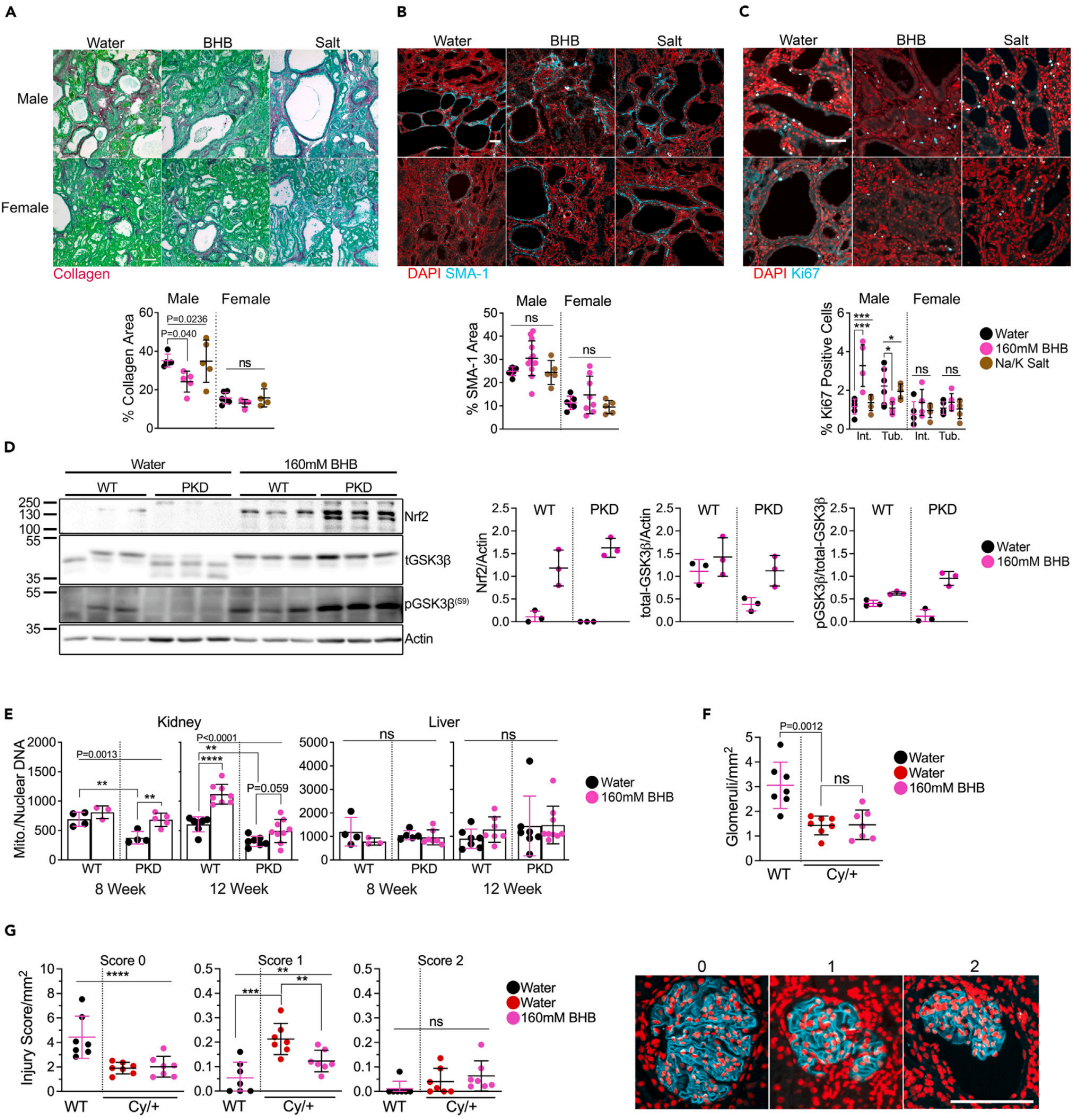
BHB ameliorates fibrosis in adult Cy/+ rats, activates preserves mitochondrial number, and increases phosphorylated-GSK-3β and Nrf2 expression (A) Sirius Red and Fast Green stain and quantification of water, 160mM BHB, and salt-supplemented male and female polycystic rats. Scale = 50μm. (B) Smooth Muscle Actin (SMA-1) immunofluorescence and quantification of water, 160mM BHB, and salt-supplemented male and female wild-type and polycystic rats. Scale = 50μm. (C) Ki67 immunofluorescence stain and quantification of water, 160m BHB, or salt-supplemented male and female wild-type and polycystic rats. Scale = 50μm. (D) Western blot and quantification of Nrf2 and total and phosphorylated GSK-3β from water and 160mM BHB supplemented male wild-type and polycystic rats. (E) Quantification of mitochondrial and nuclear DNA from kidneys and livers of water and 160Mm BHB supplemented 8-week-old and 12-week-old male wild-type and polycystic rats. (F) Number of glomeruli counted from 12-week-old male water-supplemented wild-type, water-supplemented PKD, and 160mM BHB-supplemented PKD rats. (G) (Left) Glomerular injury scoring over the measured kidney area from 12-week-old male water-supplemented wild-type, water-supplemented PKD, and 160mM BHB-supplemented PKD rats and (Right) examples of glomeruli immunostained for podocin and used for scoring rubric for quantification. Scale bar = 100μm. Rats were scored as follows. 0: No obvious morphological changes; normal. 1: Morphological change, e.g., changes in shape and structure. 2: Morphological changes as well as decreased filling of glomeruli space, increase in distance between Bowman’s capsule and podocin staining. (See also Figure S7. One-way ANOVA followed by ad hoc Tukey’s test was used for multiple comparisons. Mean and standard deviations are shown. ∗∗ = *p* < 0.01, ∗∗∗ = *p* < 0.001, ∗∗∗∗ = *p* < 0.0001).

We measured PKD outcomes and found that BHB supplementation decreased the amount of collagen deposited in male PKD kidneys (Figure 5A), non-significantly increased the amount of SMA-1 positive (Figure 5B), and increased interstitial Ki67 positive cells but decreased Ki67 in tubule cells (Figure 5C). Like fasted animals, we found that BHB treatment alone increased pGSK-3β and Nrf2 expression (Figure 5D) and appears to increase OXCT1 and BDH1 expression (Figure S7H). We measured mitochondrial DNA and found that BHB treatment increased mitochondrial DNA numbers, specifically in the kidneys of juvenile and adult PKD rats, but had no effect on mitochondrial DNA numbers in livers (Figure 5E).

To determine the effect of BHB supplementation on glomerular health, we stained male rat kidneys with podocin and measured the total number of glomeruli and the degree of glomerular injury. We observed a decrease in glomeruli between wild-type and PKD rats with no difference with BHB supplementation (Figure 5F). After scoring glomeruli, we found that BHB supplementation reduced the number of intermediately injured glomeruli compared to water alone (Figure 5G) suggesting a protective effect of BHB in preventing glomerular injury.

### BHB supplementation ameliorates PKD progression in an orthologous mouse model of PKD

**Figure 6:**
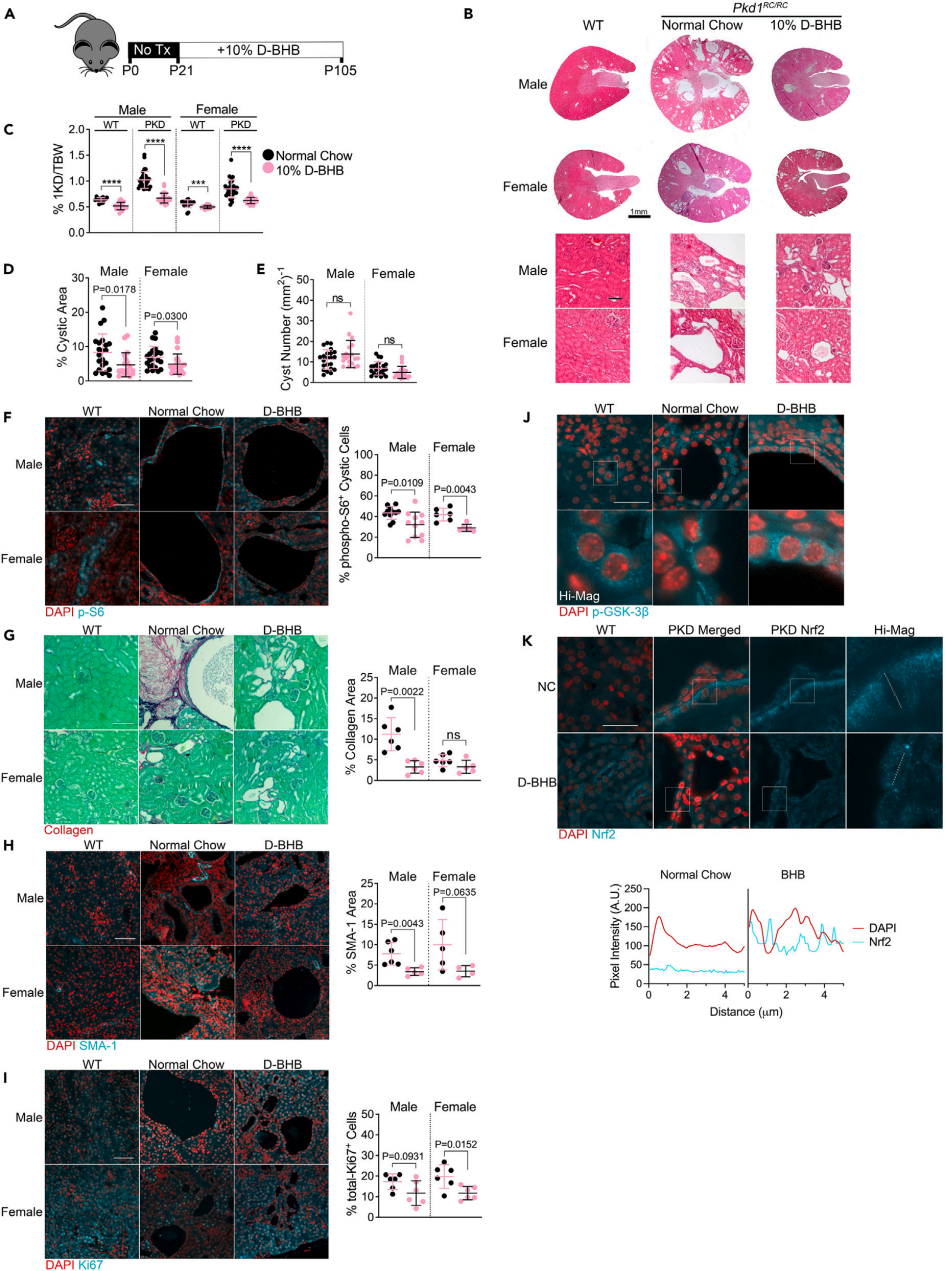
BHB supplementation ameliorates PKD progression in an orthologous mouse model of PKD (A) Schematic of experimental design for BHB administration in *Pkd1*^*+/+*^*and Pkd1*^RC/RC^ mice. (B) Hematoxylin and eosin-stained male and female mice kidneys from 3-month-old *Pkd1*^*+/+*^, *Pkd1*^RC/RC^, or *Pkd1*^RC/RC^ supplemented with D-BHB. Scale = 1mm. (C) Kidney over bodyweight of all kidneys collected from Normal Chow and D-BHB supplemented 3-month-old *Pkd1*^*+/+*^ and *Pkd1*^RC/RC^ male and female wild-type and polycystic mice. (D) Cystic area of all kidneys collected from Normal Chow and D-BHB supplemented 3-month-old *Pkd1*^*+/+*^ and *Pkd1*^RC/RC^ male and female wild-type and polycystic mice. (E) Total Cyst Number of all kidneys collected from Normal Chow and D-BHB supplemented 3-month-old *Pkd1*^*+/+*^ and *Pkd1*^RC/RC^ male and female wild-type and polycystic mice. (F) Immunofluorescence of phospho-S6^235/236^ and quantification of cytoplasmic phospho-S6^235/236^ within cystic epithelia in 3-month-old *Pkd1*^RC/RC^ male and female wild-type and polycystic mice. Scale = 50μm. (G) Sirius Red and Fast Green stain of collagen (red) and quantification from Normal Chow and D-BHB supplemented 3-month-old *Pkd1*^RC/RC^ male and female wild-type and polycystic mice. Scale = 70μm. (H) Immunofluorescence images and quantification of total Smooth Muscle Actin (SMA-1) positive area in kidneys from Normal Chow and D-BHB supplemented 3-month-old *Pkd1*^RC/RC^ male and female wild-type and polycystic mice. Scale = 50μm. (I) Immunofluorescence images and quantification of total Ki67 positive cells in kidneys from Normal Chow and D-BHB supplemented 3-month-old *Pkd1*^RC/RC^ male and female wild-type and polycystic mice. Scale = 50μm. (J) Immunofluorescence of phospho-GSK-3β^S9^ in kidneys from Normal Chow and D-BHB supplemented 3-month-old *Pkd1*^RC/RC^ male wild-type and polycystic mice. Scale = 30μm. (K) Immunofluorescence of Nrf2 in kidneys from Normal Chow and D-BHB supplemented 3-month-old *Pkd1*^RC/RC^ male wild-type and polycystic mice. Pixel intensity plot of DAPI and Nrf2 fluorescence from high-magnification panels indicated with dotted lines. Scale = 30μm. (See also Figure S8. Mann-Whitney analysis was used to compare the mean between two groups. Mean and standard deviations are shown. ∗∗∗ = *p* < 0.001, ∗∗∗∗ = *p* < 0.0001).

Until now, our experiments have utilized the non-orthologous Han rat (Cy/+) model of PKD (Figures 1, 2, 3, 4, and 5). To test whether the isomer of BHB produced during fasting, D-BHB, may show similarly beneficial effects in an orthologous model, we employed the hypomorphic *Pkd1*^*RC/RC*^ mouse model of PKD.^34^ These mice contain mutated *Pkd1* alleles*,* leading to moderately progressive PKD development, more closely mimicking human disease progression. Mice were treated with 10% D-BHB-supplemented chow for 12 weeks starting at P21 (Figure 6A). D-BHB led to a potent reduction of cystic disease progression (Figure 6B) and lowered 2-kidney-to-bodyweight ratios in both males and females (Figure 6C). The extent of the decrease in kidney mass in *Pkd1*^*RC/RC*^ mice (Figure S8A) was similar to that observed in the Cy/+ rat following fasting and BHB supplementation (Figures S2A, S3A, S5A, and S7A). D-BHB supplementation increased blood D-BHB (Figure S8B) and tended to increase glucose (Figure S8C). Like Cy/+ rats, D-BHB led to a decrease in the cystic area of *Pkd1*^*RC/RC*^ mice (Figure 6D) but without affecting cyst number (Figure 6E), suggesting that D-BHB primarily affects cyst expansion, but not cystogenesis, in this model.

Again, similar to Cy/+ rats, we found that D-BHB significantly diminished mTORC1 signaling within cystic epithelial cells (Figure 6F), decreased collagen deposition (Figure 6G), decreased the myofibroblast marker SMA-1 (Figure 6H), and reduced the proliferation marker Ki67 (Figure 6I). Similar to our Cy/+ experiments, we observed increased cytoplasmic pGSK-3β within cystic epithelial cells (Figure 6J).

We analyzed Nrf2 expression via immunofluorescence and found that PKD kidneys had significant Nrf2 expression, predominantly absent from nuclei. Using confocal microscopy immunofluorescence, we measured nuclear image slices and observed overlapping nuclear Nrf2 signal following BHB treatment (Figure 6K), suggesting that D-BHB supplementation enhances the nuclear function of the transcription factor Nrf2 in *Pkd1*^*RC/RC*^ mice.

### Parenteral BHB administration ameliorates disease progression in a juvenile orthologous mouse model

We next tested if BHB could recapitulate its beneficial effects in another, more aggressive, orthologous *Pkd1* mouse model and also asked whether the effect of BHB depends on its role as an energy substrate or as a signaling molecule. The *Pkd1-*Ksp:Cre model leads to kidney-specific deletion of *Pkd1*, causing rapid cystic disease shortly after birth.^35^ In addition to the *Pkd1*^*RC/RC*^ mouse model, with two hypomorphic Pkd1 alleles, testing the effect of BHB in a *Pkd1*-knockout can provide important insight into the mechanistic action of BHB and support for its potential efficacy.

D-BHB is the major stereoisomer produced by the liver during fasting, while L-BHB rarely forms in appreciable amounts under standard physiological conditions.^36^ Due to the stereospecificity of BDH1, L-BHB is not readily metabolized for use in the TCA cycle yet possesses many of the signaling properties of D-BHB.^37^ In our previous experiments using the Cy/+ rat, we utilized racemic BHB containing both D and L isomers (Figures 4 and 5) and D-BHB alone in the *Pkd1*^*RC/RC*^ mouse (Figure 6).

**Figure 7:**
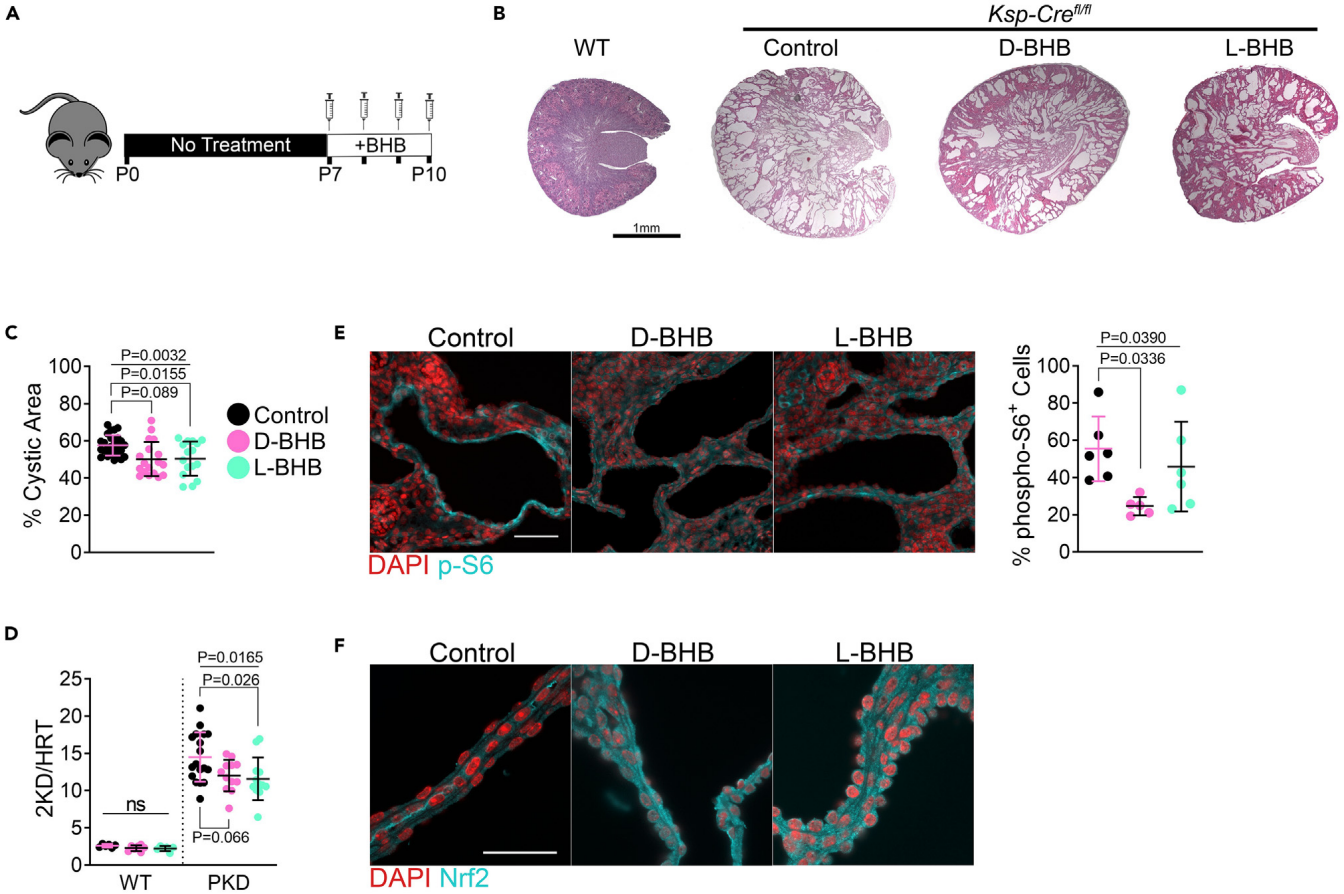
Parenteral BHB administration ameliorates disease progression in a juvenile orthologous mouse model (A) Schematic of experimental design for parenteral BHB administration in neonatal *Pkd1*-Ksp:Cre mice. (B) Hematoxylin and eosin-stained kidneys from P10 wild-type or *Pkd1*^*fl/fl*^-Ksp:Cre control, D-BHB, or L-BHB injected mice. Scale = 1mm. (C) Cystic area of all kidneys collected from P10 control, D-BHB, or L-BHB treated wild-type and *Pkd1*^*fl/fl*^-Ksp:Cre mice. (D) 2–kidney-to-heart ratio from P10 control, D-BHB, or L-BHB-treated wild-type and *Pkd1*^*fl/fl*^-Ksp:Cre mice. (E) Immunofluorescence of phospho-S6^235/236^ and quantification of cytoplasmic phospho-S6^235/236^ within cystic epithelia in P10 control, D-BHB, and L-BHB-treated *Pkd1*^*fl/fl*^-Ksp:Cre mice kidneys. Scale = 50μm. (F) Immunofluorescence of Nrf2 in P10 control, D-BHB, and L-BHB-treated *Pkd1*^*fl/fl*^-Ksp:Cre mice kidneys. Scale = 50μm. (See also Figure S9. Male and female mice were used for this experiment. One-way ANOVA followed by ad hoc Tukey’s test was used for multiple comparisons. Mean and standard deviations are shown).

To test if the effect of BHB on PKD is in part due to signaling effects, we peritoneally administered either D or L-BHB daily from postnatal day 7 to postnatal day 10 in *Pkd1-*Ksp:Cre mice (Figure 7A). Treatment with D or L-BHB isomers reduced the overall cystic phenotype (Figure 7B), leading to a significant decrease in the cystic area with L-BHB and a non-significant decrease with D-BHB (Figure 7C), this accompanied a reduction in the 2-kidney-to-heart weight ratio (Figure 7D) without significantly altering the heart size (Figures S9A and S9B). D-BHB but not L-BHB caused a reduction in the body mass of wild-type but not cystic mice (Figure S9C). BHB administration reduced mTORC1 signaling with D-BHB and, to a lesser extent, with L-BHB (Figure 7E).

We stained kidneys for Nrf2 and found that both D and L-BHB increased the expression of Nrf2 in cystic epithelia (Figure 7F). Since these effects occurred with the non-metabolizable L-BHB isoform, from these results, we conclude that BHB acts on PKD progression by mechanisms distinct from, and likely in addition to, its role as an energy source.

**Figure 8:**
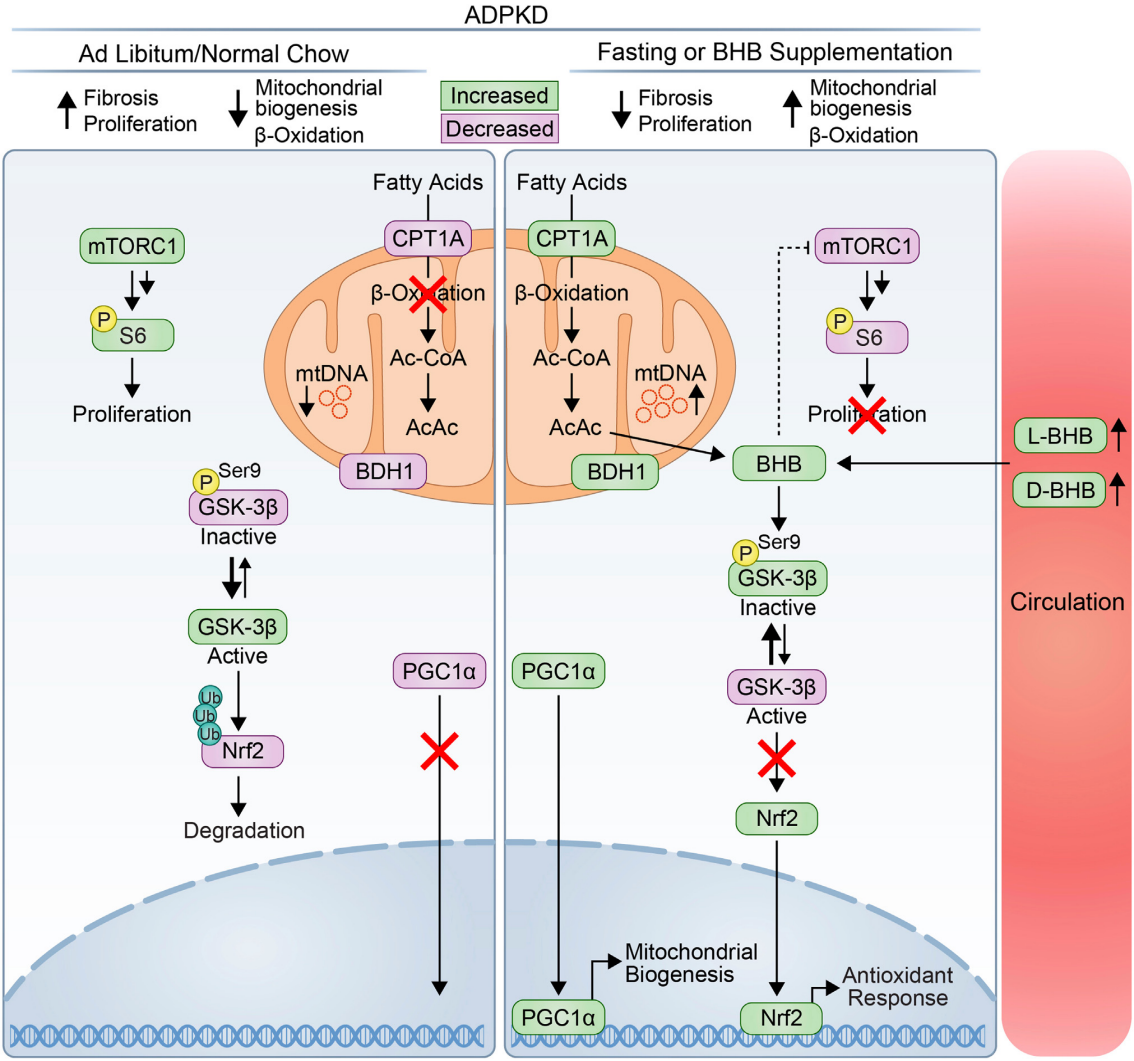
Mechanistic model of action of BHB during fasting in ADPKD mTORC1 and GSK-3beta activity are high during *ad libitum* normal chow feeding in PKD, leading to ubiquitination and degradation of Nrf2. Fasting or BHB supplementation inhibits mTORC1 activity and promotes GSK-3β phosphorylation and subsequent accumulation of Nrf2 and nuclear translocation. Additionally, fasting and BHB facilitate an increase in mitochondrial DNA number, and fatty acid oxidation and mitochondrial-associated proteins. Functionally, fasting and BHB affects PKD by decreasing fibrosis and proliferation. (Green highlighted symbols represent proteins increased, and purple highlighted symbols represent decreased proteins. Dashed line represents indirect inhibition. Multiple arrows represent multi-step activation).

Our experimental observations support a model in which ketogenic interventions lead to increased levels of BHB and that BHB putatively mediates the effects of fasting by promoting phosphorylation of GSK-3β, Nrf2 expression, and Nrf2 nuclear translocation. This in turn leads to an increase in the expression of fatty acid oxidation-associated proteins and mitochondrial number. These changes accompany improved PKD outcomes, decreasing fibrosis, mTORC1 signaling, and cellular proliferation (Figure 8).

## Discussion

We have previously demonstrated that interventions producing a ketogenic state ameliorate PKD progression can prevent and partially reverse existing renal cystic disease and that BHB can mimic these effects without altering food composition in juvenile rats.^5^ Some of our initial experiments relied heavily on the non-orthologous Han:SPRD rat (Cy/+) model of PKD, leaving the effects of ketosis related to *Pkd1* unexplored. Here, we expand on those findings, demonstrating that PF and TRF interventions effectively ameliorate and partially reverse PKD progression in juvenile and adult Cy/+ rats respectively. We also find that BHB alone recapitulates many effects of nutritional ketosis, including partial disease reversal in adult Cy/+ rats. We also demonstrate for the first time that the beneficial effect of BHB supplementation is conserved in two orthologous *Pkd1* mouse models. Using individual BHB stereoisomers, we shed light on the underlying mechanisms of BHB action, showing that BHB appears to act independently of its function as an energy substrate and alters key regulators of mitochondrial function, leading to an increase in mitochondrial-associated proteins and mitochondrial DNA copy number.

Despite the different approaches to fasting, outcomes were similar between PF and TRF. Both interventions preserved kidney function and reduced markers of advancing kidney disease, including a reversal of preexisting fibrosis. It was surprising to see that PF and TRF performed so similarly despite the dramatic difference in the application of fasting. The benefit of alternate forms of fasting demonstrates an overlapping mechanism, supporting our original hypothesis that BHB may at least partially mediate the impact of fasting. We demonstrated that BHB alone, administered in food or drinking water, produced analogous effects to fasting, including increasing GSK-3β phosphorylation and Nrf2 expression.

If BHB is a primary effector mediating the beneficial effects of KMT on renal cystic disease, what could be the underlying mechanisms? Based on our finding that the non-metabolizable stereoisomer, L-BHB, has the same effects as the rapidly metabolized D-BHB isomer, we conclude that the beneficial effects of BHB are mainly due to the signaling functions of BHB, rather than simply its role as an energy substrate.

BHB is known to have many signaling functions, many of which affect critical pathways associated with PKD progression. BHB can inhibit cAMP signaling via its receptor GPR109A,^37^^,^^38^ and cellular cAMP levels are known to be aberrantly high in PKD cyst cells, driving their proliferation.^39^ Histone deacetylase (HDAC) inhibition can ameliorate PKD progression.^40^ BHB, a class 1 HDAC inhibitor,^41^ is rapidly consumed by the heart,^42^ and inhibition of class 1 HDACs prevents cardiac hypertrophy.^43^ In support of this, we observed that Na/K salt treatment caused heart hypertrophy in male cystic rats, but was prevented by BHB. BHB increases fatty acid oxidation,^44^ which is defective in PKD cyst cells.^45^ BHB is known to inhibit the NLRP3 inflammasome,^46^^,^^47^^,^^48^^,^^49^ and chronic inflammation is a known driver of PKD progression.^50^

As an inhibitor of the NLRP3 inflammasome, BHB may affect immune cells, such as macrophages, which are known contributors to PKD progression and fibrosis.^48^ Chronic inflammation and fibrosis are features of all forms of chronic kidney disease, including PKD, with NLRP3 inflammasome activation playing a central role.^51^^,^^52^ BHB also affects signaling by increasing the posttranslational modification (e.g., β-hydroxybutyrylation and acetylation) of lysines on histones, up and downregulating critical pathways important to health and disease^53^^,^^54^ including the expression of MMP-2,^55^ a collagenase inhibited in PKD.^56^ We found that fasting and BHB treatment potently inhibited and even regressed renal collagen deposition in our experimental models. We suggest that NLRP3 inhibition plays an important role in this effect and that BHB may work in concert to modify the expression of anti-fibrotic genes.

Furthermore, we found that BHB increased the phosphorylation of pGSK-3β and Nrf2 expression. GSK-3β phosphorylates Nrf2 to allow for ubiquitination and proteasomal degradation.^57^^,^^58^^,^^59^ Previously, inhibition of GSK-3β and activation of the redox-sensitive transcription factor Nrf2 positively altered PKD progression.^17^^,^^60^^,^^61^ We found that BHB and fasting increased the phosphorylation and localization of pGSK-3β^S9^ and, similarly, Nrf2 expression and localization. Prior studies demonstrated that BHB causes inhibitory phosphorylation of pGSK-3β^S9^ and, consequently, Nrf2 accumulation.^62^ Whereas BHB increased Nrf2 expression in rats and *Ksp-Cre* mice, BHB caused Nrf2 nuclear accumulation in *Pkd1*^*RC/RC*^ mice with already high Nrf2 expression. Nrf2 is essential in response to oxidative stress and leads to the transcription of genes related to redox control and mitochondrial fatty acid oxidation. Mitochondrial structure and function loss is a known consequence of PKD in the Han rat.^10^ We observed that fasting and BHB increased the expression of the mitochondrial biogenic regulator PGC-1α and appeared to functionally increase the mitochondrial number in wild-type rats and preserve the mitochondrial number in PKD rats.

Notably, Nrf2 has been regarded as a promising drug target for ADPKD therapy, with the goal being to increase Nrf2 activity. The pharmacological Nrf2 activator bardoxolone showed promising results in early clinical trials and was then tested in the phase 3 FALCON study (NCT03918447). Unfortunately, the FALCON study terminated after bardoxolone yielded underwhelming results in a separate phase 3 trial on diabetic nephropathy, leading to the decision to discontinue the clinical development of bardoxolone. Therefore, it remains unknown what the outcome of the FALCON study would have been. Interestingly, we show that BHB supplementation potently increases Nrf2 expression. Given that BHB is an endogenous activator of Nrf2 and lacks side effects associated with pharmacological interventions, supplementation with BHB appears to be a reasonable approach in place of treatment with bardoxolone or other pharmacological compounds.

We demonstrated that BHB administration effectively reduced mTORC1 signaling via reduction in the downstream effector pS6. mTORC1 is an essential driver of PKD progression, and pharmacological mTORC1 inhibition potently inhibits PKD progression in rodent models.^27^^,^^28^^,^^29^ mTORC1 is inhibited by the tuberous sclerosis complex 2 (TSC2), which is known to associate with polycystin-1 to regulate its activity.^28^^,^^63^ TSC2 expression is upregulated in response to class 1 HDAC inhibition and is believed to be responsible for inhibiting cardiac hypertrophy.^43^ We found that D and L-BHB reduced the pS6 signal in cystic epithelia and reduced heart hypertrophy in adult animals, implicating BHB-induced HDAC inhibition as a potential mechanism. Similarly, mTORC1 inhibition with rapamycin has been shown to prevent increases in renal hypertrophy.^64^ We observed that BHB supplementation specifically reduced the size of kidneys in wild-type animals while a salt control did not. Taken together, these data imply BHB inhibits renal hypertrophy via inhibition of mTORC1 activity.

Finally, it is worth noting that activities that raise circulating BHB and promote metabolic health, such as fasting and exercise, create a signaling environment associated with improved metabolic flexibility.^65^^,^^66^^,^^67^^,^^68^ Evolution may have favored BHB as a signaling metabolite due to its intimate relationship with food scarcity, creating a highly regulated physiological signaling network dependent on periods of nutrient restriction (e.g., carbohydrate deprivation) for proper functioning. Outside of pathological conditions, BHB demonstrates metabolic flexibility as a marker of increased fatty acid oxidation and improved mitochondrial function. Overconsumption and a paucity of nutrient restriction seem to underlie many of our modern diseases of metabolism, most of which are associated with metabolic inflexibility (e.g., diabetes, cardiovascular disease, cancer, and chronic kidney disease). The activation of this nascent requirement for nutrient restriction has arisen as a viable therapeutic approach for many of these diseases.^69^^,^^70^^,^^71^ BHB supplementation may mimic nutrient restriction’s effects by simulating the metabolically flexible signaling milieu.

Given the lack of economical, safe, and effective therapeutic alternatives, ketogenic metabolic therapies, such as fasting and BHB supplementation, are very attractive as disease-modifying approaches for ADPKD. The use of fasting for well-being and religious purposes has a long history of recorded use and has been integral to the evolution of humans.^72^ In support of KMT for ADPKD, our retrospective analysis of 131 individuals with ADPKD who had implemented KMT for an average of 6 months self-reported improved renal function and beneficial effects on weight loss, hypertension, and pain.^22^ Since that report, we have contributed to the creation of a publicly accessible dietary program that is closely supervised by renal dietitians and combines a novel medical food containing BHB and citrate with the implementation of a plant-focused ketogenic diet protocol within a group setting during the course of three months. Initial results have been promising,^23^ with over 150 individuals completing the program so far. Additionally, a randomized, controlled trial that compared 3-month interventions using periodic fasting, a ketogenic diet, or a control diet was recently completed. The trial demonstrated the safety and feasibility of these dietary interventions and - surprisingly - found efficacy regarding hard kidney outcomes: eGFR based on both creatinine and cystatin-C significantly increased in the ketogenic diet arm versus control,^24^ and MRI-based total kidney volume significantly decreased in the ketogenic diet arm versus control.^25^ These early results appear extremely promising, but long-term clinical trials will be required before confident recommendations will be more widely made. Several clinical trials testing ketogenic diets in ADPKD are ongoing or planned.

The findings from this current study support our previous report on ketogenic interventions for the treatment of PKD and provide insight into the mechanism of action of KMT and an alternative or combinatorial approach using BHB supplementation alone. This study is limited by the fact it uses a non-orthologous model (Cy/+ rat) of PKD and the rapidly progressing orthologous mouse model (Ksp-Cre) that have disease progression not identical to human ADPKD. Additional clinical research will now be needed to determine if this alternative will be feasible in practice and future research efforts to determine mechanistically how BHB conveys its beneficial activity.

### Limitations of the study

This study is limited by the use of a non-orthologous rat model of PKD, the use of which may make some of the experimental evidence less applicable to human PKD. We attempted to address this limitation through inclusion of multiple rodent models of PKD, including two orthologous mouse models. We were also limited in our accessibility to large quantities of L-BHB which would have allowed us to complete the chronic feeding studies comparing the effects of BHB stereoisomers.

## Resource availability

### Lead contact

Further information and requests for resources and reagents should be directed to and will be fulfilled by the lead contact, T.W. (weimbs@ucsb.edu).

### Materials availability

This study did not generate new unique reagents.

### Data and code availability

• All data reported in this paper will be shared by the lead contact upon request.
• This paper does not report original code.
• Any additional information required to reanalyze the data reported in this paper is available from the lead contact upon request.

## Acknowledgments

The MRL Shared Experimental Facilities are supported by the MRSEC Program of the NSF under Award No. DMR 1720256; a member of the NSF-funded Materials Research Facilities Network (www.mrfn.org). We would also like to thank lab researchers that contributed genotyping and slide staining: Maria Shapiro, Charlene Brenneiser, Zirui Zeng, Han Zheng, Amy Ye, Raphael Li, Yolanna Lu, Aleena Wang, Francesca Galiani, Valentina Valdes, and Natalie Horvath.

Funding Sources: This work was supported by grants from the 10.13039/100000002NIH (R01DK109563, R01DK124895) and the US Department of Defense (W81XWH2010827), and gifts from the Amy P. Goldman Foundation and the Jarrett Family Fund to the 10.13039/100005595University of California, Santa Barbara, to support the work of T.W. T.W. has received research funding from Chinook Therapeutics, and Kyowa Kirin, and speaker fees from Otsuka.

## Author contributions

J.A.T. contributed to animal experiments, experimental design, preparation of figures, the performance of assays, and manuscript preparation. N.H. contributed to immunofluorescent and histological figures and animal experiments. D.A.A. contributed to animal experiments. B.C.K. contributed to animal experiments and UPLC analysis. T.A. contributed to experiments, quantification, and histology. M.M.H. contributed to Western blotting. E.S. contributed figure designs and Western blotting. Samantha Kruger contributed to animal experiments. S.S. contributed to animal experiments. M.F.S. contributed to animal experiments. Stella Koestner contributed to animal experiments. S.A. contributed to animal experiments. B.A.A. contributed to animal experiments and immunofluorescence. M.T. contributed to animal experiments. T.W. supervised all experiments and contributed to experimental design and manuscript preparation.

## Declaration of interests

J.A.T. and T.W. are partial owners in the Benefit Corporation Santa Barbara Nutrients and are inventors on US patent No. 11,013,705 and International Publication No. WO 2020/186154 A1 for the use of the combination of BHB and citrate in PKD. T.W. was on the scientific advisory board of Chinook Therapeutics and has received research funding from Chinook Therapeutics and Kyowa Kirin, and speaker fees from Otsuka. T.W. was on the scientific advisory board of Chinook Therapeutics.

## STAR★Methods

### Key resources table

**Table:** 

REAGENT or RESOURCE	SOURCE	IDENTIFIER
**Antibodies**		

Rabbit α-Smooth Muscle Actin	Abcam	Cat# ab5694; RRID:AB_2223021
Rabbit α-Nrf2	Thermo Fisher Scientific	Cat# PA5-27882; RRID:AB_2545358
Rabbit α-PGC1a	Novus	Cat# NBP1-04676; RRID:AB_1522118
Rabbit α-BDH1	Novus	Cat# NBP1-88673; RRID:AB_11009202
Rabbit α-OXCT1	Sigma-Aldrich	Cat# HPA012047; RRID:AB_1854857
Mouse α-CPT1α	Abcam	Cat# ab128568; RRID:AB_11141632
Mouse α-Actin	Sigma-Aldrich	Cat# A5441; RRID:AB_476744
Mouse α-Ki67, Clone B56 (RUO)	BD Biosciences	Cat# 550609; RRID:AB_393778
Rabbit α-phospho-S6 Ribosomal Protein (Ser235/236)	Cell Signaling Technology	Cat# 2211; RRID:AB_331679
Rabbit α-phospho-GSK-3β (Ser9)	Cell Signaling Technology	Cat# 9336; RRID:AB_331405
Rabbit α-Total-GSK-3β (DgC5Z) XP®	Cell Signaling Technology	Cat# 12456; RRID:AB_2636978
Goat α-rabbit HRP	Jackson ImmunoResearch Labs	Cat# 111-035-144; RRID:AB_2307391
Goat α-mouse HRP	Jackson ImmunoResearch Labs	Cat# 115-035-044; RRID:AB_2338503
Goat α-rabbit AlexFluor 594	Thermo Fisher Scientific	Cat# A-11012; RRID:AB_2534079
Goat α-mouse AlexaFluor 594	Thermo Fisher Scientific	Cat# A-11005; RRID:AB_2534073
Rabbit α-Podocin	Thermo Fisher Scientific	Cat# PA5-79757; RRID:AB_2746872

**Chemicals, peptides, and recombinant proteins**		

Sodium D-BHB and L-BHB	Shaoxing King-Year Biochemical Co., Ltd	CAS ID: 300-85-6
PicoLab Rodent Diet 20	Lab Diet	Cat#5053
D/L-β-hydroxybutyrate, KetoForce	Ketosports	
Zymo-spin TM IICR column mini-prep	Zymo Research	Cat# D4068
Sirius Red/Fast Green collagen staining kit	Chondrex	Cat# 9046
IGF-1 Rat ELISA Kit	Invitrogen	Cat# ERIGF1
D-beta-hydroxybutyric acid (D-BHB)	Sigma Aldrich	CAS:625-72-9; Cat# 54920
L-beta-hydroxybutyric acid (L-BHB)	Sigma Aldrich	CAS:6168-83-8; Cat# 54925
Sodium d_4_-DL-beta-hydroxybutyrate (Na d_4_-DL-BHB)	Cayman Chemical	CAS:1219804-68-8; Cat# 14158
S-1-(2-pyrrolidinylmethyl)-pyrrolidine (S-PMP)	Sigma Aldrich	CAS:51207-66-0; Cat# P12411G
triphenylphosphine (TPP)	Sigma Aldrich	CAS:603-35-0; Cat# 140420250
2,2’-dipyridyl disulfide (DPDS)	Sigma Aldrich	CAS:2127-03-9; Cat# D11145G
LCMS Grade Water with 0.1% Formic Acid (v/v)	Fisher Scientific	CAS:7732-18-5; 64-18-6; Cat# LS118-4
LCMS Grade Acetonitrile with 0.1% Formic Acid (v/v)	Fisher Scientific	CAS:75-05-8; 64-18-6; Cat# LS120-4
LCMS Grade Acetonitrile	Fisher Scientific	CAS:75-05-8; Cat# A995-4
LCMS Grade Methanol	Fisher Scientific	CAS:67-56-1; Cat# A456-4
MilliQ Water	Fisher Scientific	CAS:7732-18-5
Phosphatase inhibitor cocktail 2 & 3	Sigma-Aldrich	Cat# P5726; Cat# P0044
pepstatin E110	Chemicon	CAS:26305-03-3
leupeptin E18	Chemicon	CAS:103476-89-7
antipain E13	Chemicon	CAS:149116-08-5
Benzamidine	Sigma-Aldrich	CAS:618-39-3
Traysol	Bayer	CAS:9087-70-1

**Critical commercial assays**		

QuantiChrom creatine assay kit	BioAssay Systems	Cat# DICT-500
Precision Xtra	Abbott	Cat# 9881465
Contour Next EZ	Bayer	Cat# 193725201

**Experimental models: Organisms/strains**		

"*Ksp-Cre*" Mice, B6.Cg-Tg(Cdh16-cre)91Igr/J	Jackson Laboratory	JAX:012237; MGI:4412080
*Pkd1^RC/RC^* Mice, p.Arg3277Cys	University of Augusta, Augusta, Georgia	
Han Rat, Anks6^PKD^		RRID:RGD_11535000

### Experimental model and study participant details

#### Animal studies

All animal studies were performed with the approval of the University of California Santa Barbara Institutional Animal Care and Use Committee and conform to all standards of animal care and well-being. All animals were housed in the animal resource center at the University of California Santa Barbara using a 12-hour light/dark cycle at 74°F with *ad libitum* access to food, water, and enrichment unless otherwise specified. Rats and mice were weaned at postnatal day 21, separated by sex, group-housed, and randomly assorted, irrespective of genotype. Food and water intake were measured throughout all experiments, with animal weights measured weekly. β-hydroxybutyrate was measured using a blood meter (Precision Xtra; Abbott) or UPLC. A glucometer measured blood glucose (Contour Next EZ; Bayer). Animals were anesthetized using a combination of 200mg:20mg/kg ketamine:xylazine, followed by cervical dislocation before tissue removal. Tissue samples were snap-frozen in liquid nitrogen following removal for later analysis. Serum samples were collected via cardiac puncture, transferred to a Microtainer tube (Cat# B-D365967; BD), separated by centrifugation, and snap-frozen in liquid nitrogen. Animals were sacrificed at ∼9 AM for experimental consistency. The lead researcher was not blinded to the genotypes of animals in treatment groups. Researchers performing daily maintenance were not aware of experimental genotypes.

##### Food

Mice and rats were fed “Normal Chow” (Rodent Diet 20, 5053; PicoLab®) containing ∼20% protein, ∼4.5% fat, 0.64% P, 0.22% Mg, 1.10% K, and 0.30% Na.

##### Periodic fasting

Weekly 48-hour fasts were performed in 3-week-old rats once a week for five weeks (Juvenile model) or 8-week-old rats for four weeks (Adult model), followed by five days of *ad libitum* feeding. BHB and glucose measurements were recorded in adult rats at the end of the fasting period.

##### Time-restricted feeding

Rats on time-restricted feeding schedules were performed in either 3-week-old rats for five weeks (Juvenile model) or 8-week-old rats for four weeks (Adult model). *Ad libitum* access to food was provided for 8 hours a day, followed by 16 hours of food restriction. Juvenile feeding Z0 occurred during the animal’s dark period, whereas adult Z0 feeding occurred during the light period (Figures 1A and 2A, respectively).

##### Pkd1^RC/RC^ mice

3-Week-old male and female RC/RC mice were supplemented with 10% sodium D-BHB (Shaoxing King-Year Biochemical Co., Ltd or “D-Max” by Julian Bakery. Both are from the same supplier.) mixed by Envigo using a NaCl depleted starting diet to reduce overall sodium consumption. This formula is part of a series TD.200319 - TD.200322 designed with a similar composition. This diet adds 118g D-sodium ꞵ-hydroxybutyrate salt per kg to create a diet with 10% D-ꞵ-hydroxybutyrate. Approximate nutrient content of the diet contains 19% protein, 5% fat, 0.6% P, 0.15% Mg, 0.8% K, and 2% Na. A blue food coloring was added for visual distinction.

##### Pkd1-Ksp:Cre mice

Ksp-Cre mice (ID B.6.Cg-Tg(Cdh16-cre)91Igr; Jackson Laboratory) were crossed with *Pkd1*^*fl/fl*^ mice (received as a gift from Augusta University). The Sodium D or L-BHB (Shaoxing King-Year Biochemical Co., Ltd) was dissolved in sterile PBS and sterile-filtered with a 0.2-micron syringe filter before administration with a pH of ∼6.5. Neonate male and female wild-type and polycystic mice were treated intraperitoneally with 15μmol/g daily using a 1M D-BHB or L-BHB solution from P7 until P10 (4 injections). Controls were treated with an equivalent volume of PBS.

##### Adult BHB-supplemented rats

A racemic sodium/potassium salt of D/L-β-hydroxybutyrate (KetoForce; Ketosports) was added to water and provided *ad libitum* to 8-week-old rats for BHB studies with a concentration of 4.2% (approximately 160mM). Control rats received equimolar sodium/potassium chloride salts in place of BHB.

##### Creatinine

Serum creatinine was calculated using a QuantiChrom creatine assay kit (Cat# DICT-500; BioAssay Systems).

**Table:** Animals used for experiments

Experiment	Male n (WT/PKD)	Female n (WT/PKD)
*8-Week Time-Restricted Feeding*	13/8	8/12
*12-Week Time-Restricted Feeding*	7/9	N/A
*8-Week Periodic Fasting*	8/11	4/5
*12-Week Periodic Fasting*	19/12	11/9
*12-Week BHB Water Treated*	12/12	12/12
*12-Week Salt Treated*	7/8	6/9
*RC/RC BHB Mice*	18/16	10/16
*RC/RC Mice Controls*	18/18	8/15
*Ksp-Cre D-BHB Injections*	2/6	6/7
*Ksp-Cre L-BHB Injections*	2/5	4/7
*Ksp-Cre Controls*	5/10	1/10
*8-Week Rat Controls*	10/10	10/11
*12-Week Rat Controls*	8/10	10/11

### Method details

#### Immunofluorescence

Deparaffinized slides were rehydrated to TBS, subjected to pressure cooker antigen retrieval with 10mM sodium citrate pH 6.0 for 20 minutes, then cooled with running tap water over the cooker and flooding the cooker once cooled. Slides were washed in TBST and then blocked (1% BSA, 0.1% TX-100, 0.1% fish skin gelatin in TBST) in a humid chamber at 37°C for 60 minutes. Primary antibodies were mixed with blocking buffer and incubated on sections overnight at 4°C. Slides were washed in TBST, incubated in 0.1% Sudan Black B in 70% ethanol for 20 minutes, and jet washing with TBST. Slides were then incubated with secondary antibody in a humid chamber protected from light for 60 minutes at 37°C. Slides were then washed in TBST, fixed with 10% NBF for 10 minutes, washed in TBST and stained with DAPI in TBS for 10 minutes, rinsed in TBST, and mounted using Prolong Gold (Cat# P36930; ThermoFisher).

#### Histology

Kidney tissue samples were excised from rats and immediately rinsed in PBS, then weighed, cut medially, and fixed in 10% neutral buffered formalin for 24 hours at ambient temperature, followed by paraffinization with 2x 1 hour immersion in 70% ethanol, 1 hour in 95% ethanol, 0.5 hours in 95% ethanol, 0.5 hours in 100% ethanol, 1 hour in 100% ethanol, 2 hours in 100% ethanol, 1 hour in xylene, 2x 1.5 hours in xylene, 1.5 hours and 3 hours in paraffin wax at 60°C. Kidneys were cut into 5μm sections on Superfrost Plus slides (Cat# 12-550-15; Fisher Scientific) for all histology and immunofluorescence applications. Samples were deparaffinized in 2 changes of xylenes for 5 minutes and rehydrated in decreasing alcohol series (2x 100%, 2x 95%, and 70%) prior to histological or immunofluorescence applications.

##### Hematoxylin and Eosin

Rehydrated samples were placed in deionized water, then put in hematoxylin solution for 1 minute, rinsed in running tap water, then placed into eosin for 45 seconds and dipped ten times in 2x 95% and 2x 100% ethanol before being placed into two changes of xylenes for 5 minutes each time and finally mounted using Permount (Cat# SP15-100; Fisher Scientific).

##### Collagen

Deparaffinized kidney samples were stained using a Sirius Red/Fast Green collagen staining kit (Cat# 9046; Chondrex).

#### Mitochondrial number qPCR

Zhang et al.^73^ previously described quantifying mitochondrial DNA copy number. DNA was extracted from approximately 25mg of frozen tissue using a Zymo-spin TM IICR column mini-prep (Cat# D4068; Zymo Research) following the manufacturer's instructions. 30μL of isolated DNA was then purified by adding 5μl of 3M sodium acetate (pH 5.2) and 150μl of ice-cold 100% ethanol, mixed by inversion, and stored overnight at -20°C. Samples were then pelleted at 4°C for 25 minutes, decanted and washed with 200μL 70% ethanol, air-dried, and resuspended in sterile TE buffer. Samples were then diluted to 3ng/μl in TE and subjected to qPCR using 1μl of DNA template (Cat# 6020; Promega).

##### Primers used

195bp product Rat Clusterin (TRPM-2).

**Table:** 

Forward 5’	GGTGTACTTGAGCAGAGCGCTATAAAT	3’
Reverse 5’	CACTTACCCACGGCAGCTCTCTAC	3’

235bp product Rat Mitochondria (Cytochrome B).

**Table:** 

Forward 5’	CCTCCCATTCATTATCGCCGCCCTTGC	3’
Reverse 5’	GTCTGGGTCTCCTAGTAGGTCTGGGAA	3’

PCR conditions were as follows: 1 Cycle of 94°C for 5 minutes, 35 Cycles of 94°C for 15 secs, 63°C for 45 secs, 72°C for 60 secs, then one cycle of 94°C for 60 secs, one cycle of 72°C for 30 secs, and one cycle of 95°C for 30 secs.

The mitochondrial number was determined by obtaining CT values for mitochondrial and nuclear DNA and then calculating ΔCT=(nucDNA CT - mtDNA CT). The relative mitochondrial number was calculated using (mitochondrial number = 2x2^ΔCT^).

#### Western blotting

Approximately 10mg of snap-frozen tissue samples were lysed in 200μL of SDS lysis buffer (4% SDS, 100mM Tris HCl pH 6.8, 20% glycerol, 1:1000 protease inhibitor cocktail (pepstatin E110, leupeptin E18, antipain E13; Chemicon, Benzamidine; Sigma-Aldrich, Trasylol; Bayer) and 1:100 phosphatase inhibitor cocktail 2 & 3 (Cat# P5726 and P0044; Sigma-Aldrich) followed by 10 minutes of heating at 100°C. Samples were diluted to 2% SDS, and 25μg of the sample was loaded for SDS-PAGE. Transfer onto nitrocellulose membrane occurred on ice at 100V for 90 minutes, then blocked using 5% BSA-TBST for phospho antibodies and 5% milk-TBST for non-phospho antibodies for 20 minutes at 37°C. Membranes were incubated overnight with primary antibodies in either 1% BSA-TBST or 1% milk-TBST corresponding to blocking conditions. Membranes were incubated for 1 hour with 1:10k secondary antibody in either 1% BSA-TBST or 1% milk-TBST corresponding to blocking conditions. Membranes were then imaged using an Azure 600 (Azure Biosystems).

#### ELISA

For IGF-1 quantification, an enzyme-linked immunosorbent assay (ELISA) kit (Cat# ERIGF1; Invitrogen) was used. Serum samples from adult rats were diluted and analyzed according to the manufacturer's instructions.

#### UHPLC mass spectrometry

L and D BHB isomers were measured in serum using Ultra-High-Pressure Liquid Chromatography Mass Spectrometry (UHPLC-MS) following derivatization to separate isomers. The full methods are outlined in the supplemental methods.

##### Chemicals and materials

D-beta-hydroxybutyric acid (D-BHB) (CAS:625-72-9, Cat# 54920, Sigma Aldrich).

L-beta-hydroxybutyric acid (L-BHB) (CAS:6168-83-8, Cat# 54925, Sigma Aldrich).

Sodium d_4_-DL-beta-hydroxybutyrate (Na d_4_-DL-BHB) (CAS:1219804-68-8, Cat# 14158, Cayman Chemical).

S-1-(2-pyrrolidinylmethyl)-pyrrolidine (S-PMP) (CAS:51207-66-0, Cat# P12411G, Sigma Aldrich).

triphenylphosphine (TPP) (CAS 603-35-0, Cat# 140420250, Sigma Aldrich).

2,2’-dipyridyl disulfide (DPDS) (CAS:2127-03-9, Cat# D11145G, Sigma Aldrich).

##### Solvents

LCMS Grade Water with 0.1% Formic Acid (v/v) (CAS:7732-18-5, 64-18-6, Cat# LS118-4, Fisher Scientific).

LCMS Grade Acetonitrile with 0.1% Formic Acid (v/v) (CAS:75-05-8, 64-18-6, Cat# LS120-4, Fisher Scientific).

LCMS Grade Acetonitrile (CAS:75-05-8, Cat# A995-4, Fisher Scientific).

LCMS Grade Methanol (CAS:67-56-1, Cat# A456-4, Fisher Scientific).

MilliQ Water (CAS:7732-18-5, Millipore Sigma).

##### Materials

96-well 0.2um polypropylene vacuum filtration plate (Cat# PI90036, ThermoScientific).

###### Software

MassLynx (V4.1, Waters Inc.) was used for both the acquisition and processing of data.

The following methods were modified from a previously published method (Tsutsui, H. et al., 2012).

###### UPLC-ESI-QToF

A Waters Acquity H-class Ultra-High-Pressure Liquid Chromatography (UPLC) system coupled to a Waters Xevo G2-XS QToF Quadrupole Time-of-Flight Mass Spectrometer was used for the analysis, and the column employed was a Waters BEH C18 UPLC column (1.7 μm, 100 x 2.1 mm). The following parameters were used for the analysis:

##### UPLC parameters

Injection Volume: 1.00 μL

Column Temperature: 40°C

Flow Rate: 0.350 mL/min

##### Gradient profile

0.00 min – 100% H_2_O-FA; 0% ACN-FA

5.00 min – 90% H_2_O-FA; 10% ACN-FA

8.00 min – 0% H_2_O-FA; 100% ACN-FA

9.00 min – 95% H_2_O-FA; 5% ACN-FA

11.01 min – 100% H_2_O-FA; 0% ACN-FA

Autosampler Temperature: 10°C

##### QToF parameters

Polarity: positive

Analyzer: sensitivity mode

Capillary Voltage: 0.50 kV

Sampling Cone Voltage: 30 V

Source Temperature: 120°C

Source Offset: 80

Desolvation Temperature: 350°C

Cone Gas Flow: 50 L/hr

Desolvation Gas Flow: 1000 L/hr

LM Resolution: 10

HM Resolution: 15

Sample Infusion Flow Rate: 10 μL/min

###### Standard solutions

D-BHB and L-BHB were made at 1,000.00 mg/L in ACN. The internal standard Na-d4-DL-BHB was made at 10.00 mg/L in a solution of 90:10 ACN:MilliQ water (v/v), and Na-DL-AHB was made at 1,000.00 mg/L in the same 90:10 solution. The reagents S-PMP, TPP, and DPDS were each made to 20.00 mM in ACN.

###### Working solutions

D-BHB and L-BHB were made through 10-fold dilutions of the standards with ACN to make solutions with concentrations of 100.00 mg/L, 10.00 mg/L, 1.00 mg/L, and 0.10 mg/L.

###### Calibration solutions

The calibration range for D-BHB and L-BHB was from 1 ug/L – 1000 ug/L. To a microcentrifuge tube was added an appropriate aliquot of the analyte, 100.0 μL of internal standard, 100.0 μL of S-PMP, 100.0 μL of TPP, 100.0 μL of DPDS, and then diluted with the appropriate amount of ACN in order to reach a final volume of 1.00 mL. The solution was then vortexed and allowed to react at room temperature overnight to ensure a complete reaction. Once reacted, 100.0 μL of calibration solution was transferred to an LCMS vial and diluted with 900. μL of a solution of 98:1.6:0.4 H_2_O-FA:MeOH:ACN (v/v/v). The LCMS vial was then vortexed and loaded into the instrument for analysis. Calibration solutions were made in triplicate.

###### Sample preparation

To a microcentrifuge tube was added 10.00 μL of sample (serum or urine), 100.0 μL of internal standard, and 890. μL of ACN. The tube was then vortexed, and centrifuged for 5 minutes at 15,000 rpm, and the supernatant was filtered through a 96-well 0.2 μm polypropylene vacuum filtration plate. The filtered sample was then transferred to a microcentrifuge tube, and the solvent was evaporated in a vacuum centrifuge at 40°C. To the remaining residue was added 700. μL of ACN, 100.0 μL of S-PMP, 100.0 μL of TPP, and 100.0 μL of DPDS. The solution was then vortexed and allowed to react at room temperature overnight to ensure a complete reaction. Once reacted, 100.0 μL of sample solution was transferred to an LCMS vial and diluted with 900. μL of 98:1.6:0.4 H_2_O-FA:MeOH:ACN (v/v/v). The LCMS vial was then vortexed and loaded into the instrument for analysis.

###### Calibration curve

Calibration curves were constructed for both D-BHB and L-BHB by plotting the average peak area ratios of D-BHB and L-BHB to the internal standard against the corresponding concentration of analyte, and then using the method of least squares linear regression to compute the equations of the lines. The calibration was acceptable with an R^2^ value ≥ 0.995.

###### Quantification of D-BHB and L-BHB from samples

The concentrations of D-BHB and L-BHB from the samples were calculated by determining the peak area ratios of D-BHB and L-BHB to the internal standard then using the equations of the lines to determine the corresponding concentration.

### Quantification and statistical analysis

#### Blinding

Researchers performing quantification were blinded and provided animal ID numbers without knowledge of specific treatments.

#### Fibrosis

Sirius red-stained sections were imaged at 100x magnification for quantification. Ten images from cortical regions were taken from each kidney. Using Photoshop (Adobe), a grid was placed over each image, and intersections with Sirius red stain were counted as positive, with intersections overlaid on negative space or outside of tissue excluded from the total number of potential intersections. The total number of positive intersections was then divided by the total possible number of intersections to obtain the percent of fibrosis.

#### Rat cystic index

Hematoxylin and eosin sections were imaged at 100x magnification for quantification. Ten images from cortical regions were taken from each kidney. Using Photoshop (Adobe), a grid was placed over each image, and intersections overlaid on cysts were counted as positive, with other intersections counted as negative, with space intersecting outside of the tissue excluded from the total number of potential intersections. The total positive intersections were divided by the possible intersections to obtain the cystic index.

#### Rat cyst number and cyst size

Whole kidney sections were imaged using a dissecting scope, and individual cysts were manually counted using the wand tool in FIJI (ImageJ).

#### Pkd1^RC/RC^ cystic index, cyst size and cyst number

Full kidney sections were created from 40x stitched images, and individual cysts were counted with FIJI (ImageJ)^74^ using the wand tool. The total area of cysts counted was divided by the total kidney area to obtain the cystic area. Wild-type kidneys were similarly counted to determine the cutoff size for cysts in polycystic mice. Due to the heterogeneous phenotype in these mice, both kidneys were counted from each animal when possible.

#### Pkd1-Ksp:Cre cystic index

Full kidney sections were created from 40x stitched images, and cysts were counted using the analyze particle feature in FIJI (ImageJ).^74^ Images were modified with the threshold function, and the total white space was counted and divided by the total kidney area to determine the cystic area.

#### Smooth muscle actin

Smooth muscle actin-stained sections were imaged at 200x magnification for quantification. Ten images from cortical regions were taken from each kidney. Using Photoshop (Adobe), a grid was placed over each image, and intersections with smooth muscle actin stain were counted as positive and other intersections counted as negative, with intersections overlaid on negative space excluded from the total number of potential intersections. The total number of positive intersections was divided by the total possible number of intersections to obtain the percent of smooth muscle actin positive area.

#### pS6

pS6^S235/236^ stained sections were imaged at 200x magnification for quantification. Five images from cortical regions were taken from each kidney. Cells with cytoplasmic staining were considered positive for pS6. All cysts/dilated tubules from each image were quantified by counting positive cells within each cyst/tubule and dividing the number of positive cells over the total number of cells in each cyst/tubule.

#### Ki67

Ki67 stained sections were imaged at 200x magnification for quantification. Five images from cortical regions were taken from each kidney, with approximately 2,000 cells counted for each animal. Cell number was determined with DAPI positive images using FIJI (ImageJ)^74^ to automate cell counting. Ki67-positive cells were manually counted and classified by location as interstitial (existing outside of tubules and cysts) or tubular (existing in cysts or tubules) for rat experiments, and total-Ki67-positive cells were counted for mice experiments. The number of positive cells from all five images for each location was divided by the number of cells counted to obtain the percentage of Ki67 positive cells.

#### Nrf2 localization

High magnification (600x) images of *Pkd1*^*RC/RC*^ mice were obtained, and a single confocal slice in which nuclei were maximally visible was used to determine Nrf2 localization. Using FIJI (ImageJ),^74^ a composite RGB image of Nrf2 and DAPI was created, a line was drawn through the nucleus, and a plot profile was obtained using the RGB Profiles Tool macro.

#### Glomerular health scoring

Kidney sections were stained for podocin images at 400x magnification, and all glomeruli were scored using the following rubric as previously described:^75^

0: No obvious morphological changes; normal

1: Morphological change, e.g., changes in shape and structure

2: Morphological changes, as well as decreased filling of glomeruli space, increase in distance between Bowman's capsule and podocin

#### Statistics

Non-parametric two-tailed Mann-Whitney analysis was used to compare the differences between 2 means where applicable, and one-way ANOVA analysis followed by ad hoc Tukey’s test was used to compare differences between means of groups using Prism 8 (GraphPad). Individuals were used as the experimental unit for analysis. Cage cohorts were used for food and water intake analysis. In analyses that use fewer than the entire group of experimental animals, animals were chosen randomly for inclusion in the analysis. A minimum of 3 experimental replicates were used for all experiments. Multiple litters were used for each experimental condition tested to avoid potential litter biases.
