# Supplementary material for: β-hydroxybutyrate recapitulates the beneficial effects of ketogenic metabolic therapy in polycystic kidney disease
Source: iScience. 2024 Aug 20;27(9):110773. doi: 10.1016/j.isci.2024.110773 (PMC11418134; doi:10.1016/j.isci.2024.110773)
Supplement: Document S1. Figures S1–S9 [file mmc1.pdf]

ISCI, Volume *27*

Supplemental Information

β-hydroxybutyrate recapitulates the beneficial effects of ketogenic metabolic therapy in polycystic kidney disease

Jacob A. Torres, Nickolas Holznecht, David A. Asplund, Bradley C. Kroes, Tselmeg Amarlkhagva, Matthias M. Haeffner, Elizabeth H. Sharpe, Stella Koestner, Sebastian Strubl, Margaret F. Schimmel, Samantha Kruger, Shagun Agrawal, Brina A. Aceves, Muthusamy Thangaraju, and Thomas Weimbs

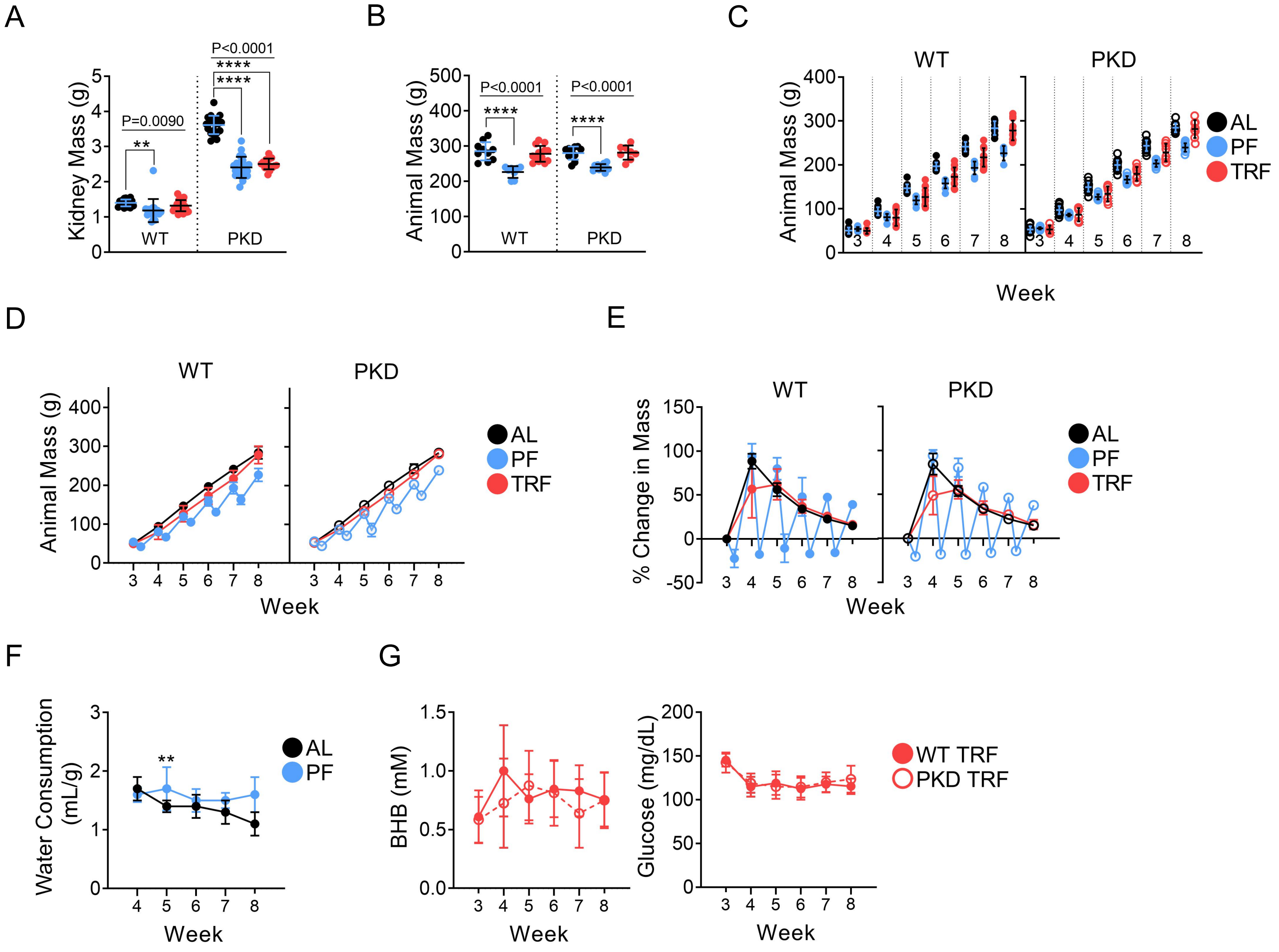

Supplemental Figure 1

Supplemental Figure 1: *Animal Measurements of Periodic Fasting and Time-Restricted Feeding Juvenile Male Rats, Related to Figure 1*

A) Kidney masses of 8-week-old ad libitum, PF and TRF male wild-type and polycystic rats. B) Animal masses of 8-week-old ad libitum, PF and TRF male wild-type and polycystic rats. C) Weekly animal masses of individual ad libitum, PF and TRF male wild-type and polycystic rats. D) Weekly average change in animal masses of ad libitum, PF and TRF male rats. E) Percentage change in animal mass of ad libitum, PF and TRF male rats. F) Weekly water consumption of ad libitum and periodic fasting male rats. G) Blood BHB and blood glucose in time-restricted male rats.

(One-way ANOVA followed by ad hoc Tukey's test was used for multiple comparisons. Mann-Whitney analysis was used to compare the means between groups. Mean and standard deviations are shown. **=P<0.01, ****=P<0.0001)

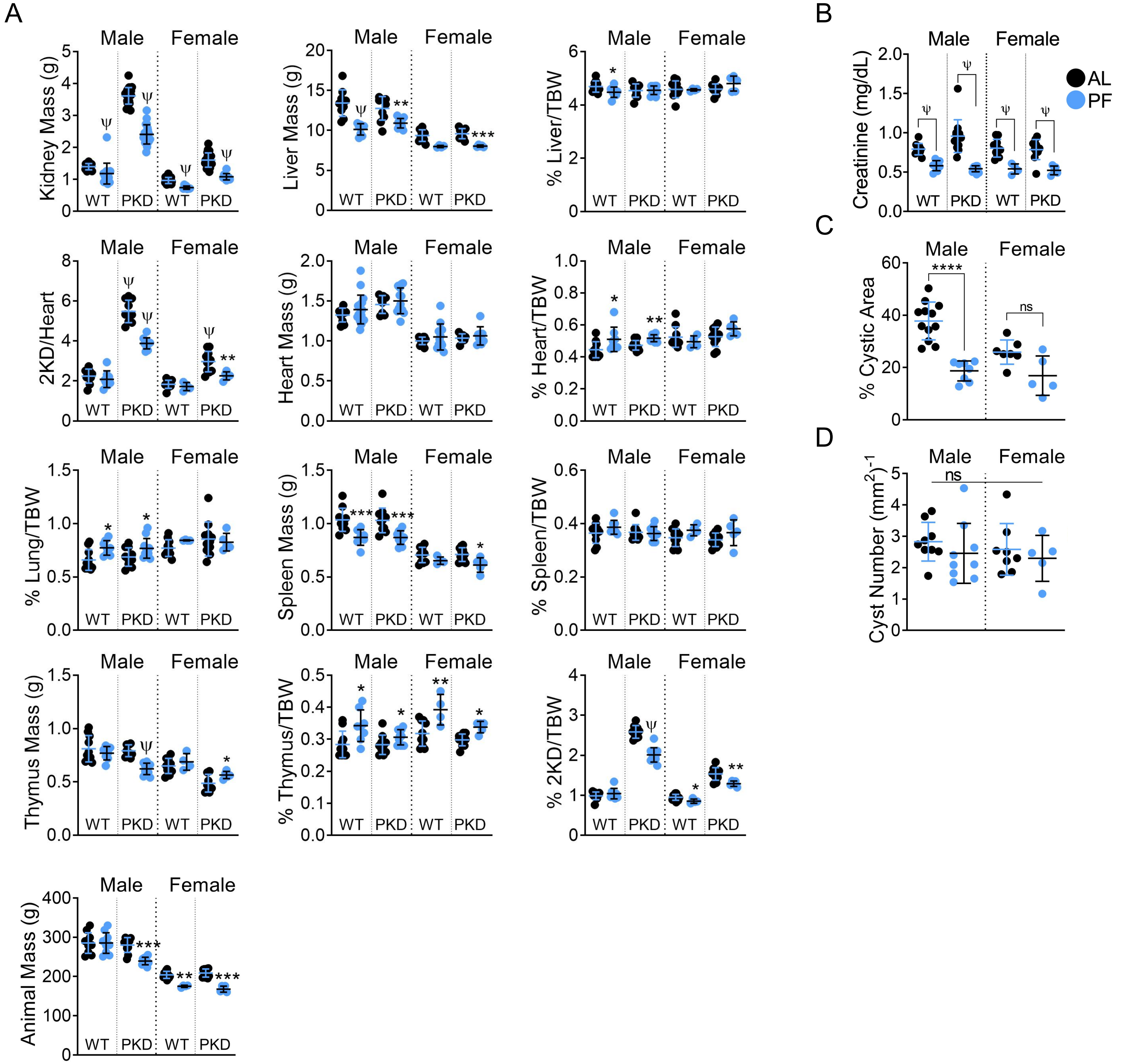

Supplemental Figure 2

Supplemental Figure 2: *Animal Measurements of Periodic Fasting Juvenile Male and Female Rats, Related to Figure 1*

A) Tissue masses of 8-week-old ad libitum and periodic fasting male and female wild-type and polycystic rats. B) Serum creatinine from 8-week-old ad libitum and periodic fasting male and female wild-type and polycystic rats. C) Cystic area of 8-week-old ad libitum and periodic fasting male and female wild-type and polycystic rats. D) Cyst Number per mm^2 from ad libitum and periodic fasting male and female wild-type and polycystic rat kidneys.

(One-way ANOVA followed by ad hoc Tukey's test was used for multiple comparisons. Mann-Whitney analysis was used to compare the mean between two groups. Mean and standard deviations are shown. *=P<0.05, **=P<0.01, ***=P<0.001, ****/ψ=P<0.0001)

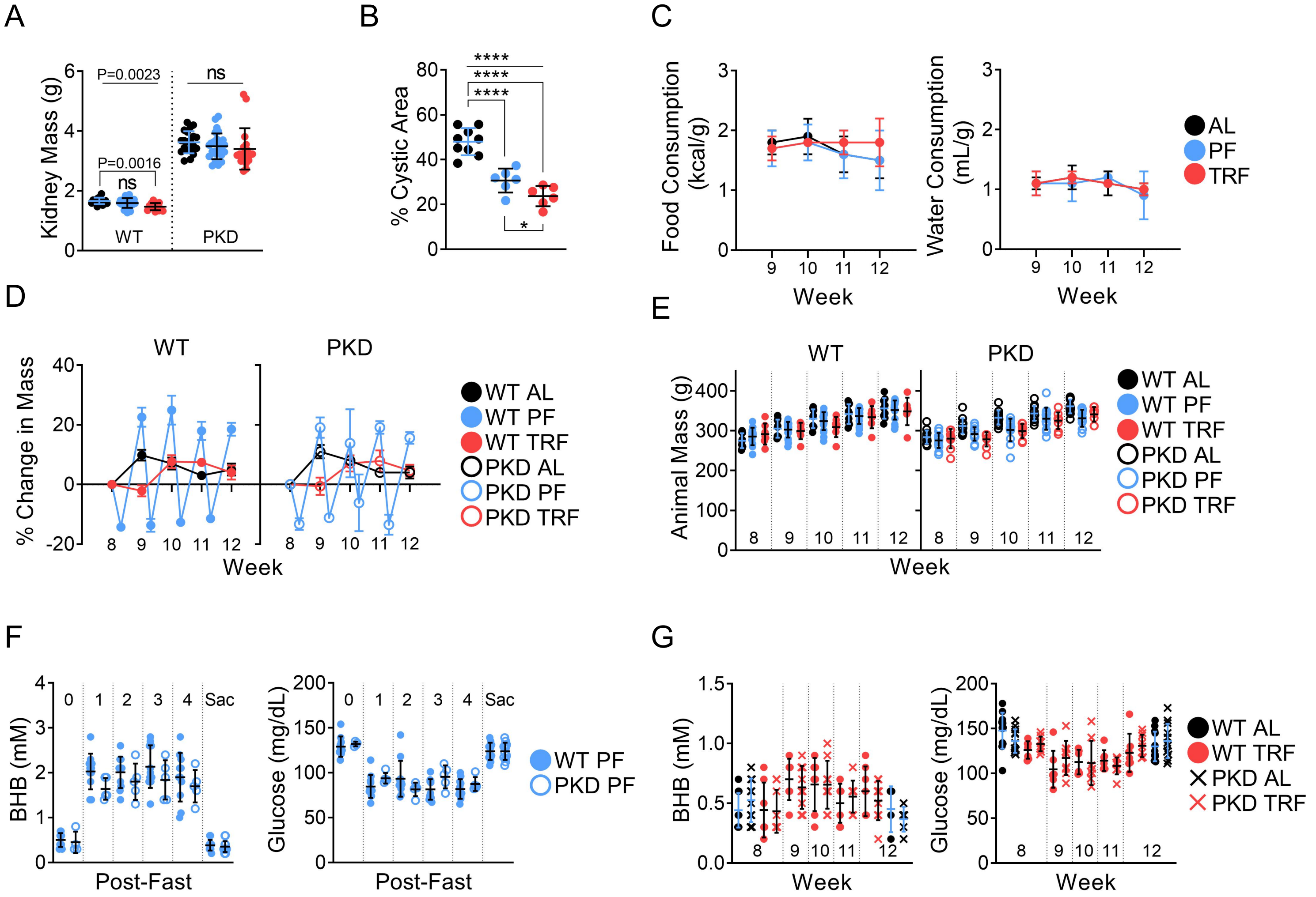

Supplemental Figure 3

Supplemental Figure 3: *Animal Measurements of Periodic Fasting and Time-Restricted Feeding Adult Male Rats, Related to Figure 2 and Figure 3*

A) Kidney masses from 12-week-old ad libitum, PF and TRF male wild-type and polycystic rats. B) Cystic area of 12-week-old ad libitum, PF and TRF male polycystic rat kidneys. C) Weekly food and water consumption of ad libitum, PF and TRF male rats. D) Percentage change in animal mass of ad libitum, PF and TRF male rats. E) Weekly animal masses of ad libitum, PF and TRF male wild-type and polycystic rats. F) Weekly pre-fast (T=0), post-fast, and at time of sacrifice blood BHB and blood glucose in periodic fasting male wild-type and polycystic rats. Sac=Sacrifice on P84

G) Weekly blood BHB and blood glucose in time-restricted male wild-type and polycystic rats.

(One-way ANOVA followed by ad hoc Tukey's test was used for multiple comparisons. Mean and standard deviations are shown. *=P<0.05, **=P<0.01, ***=P<0.001)

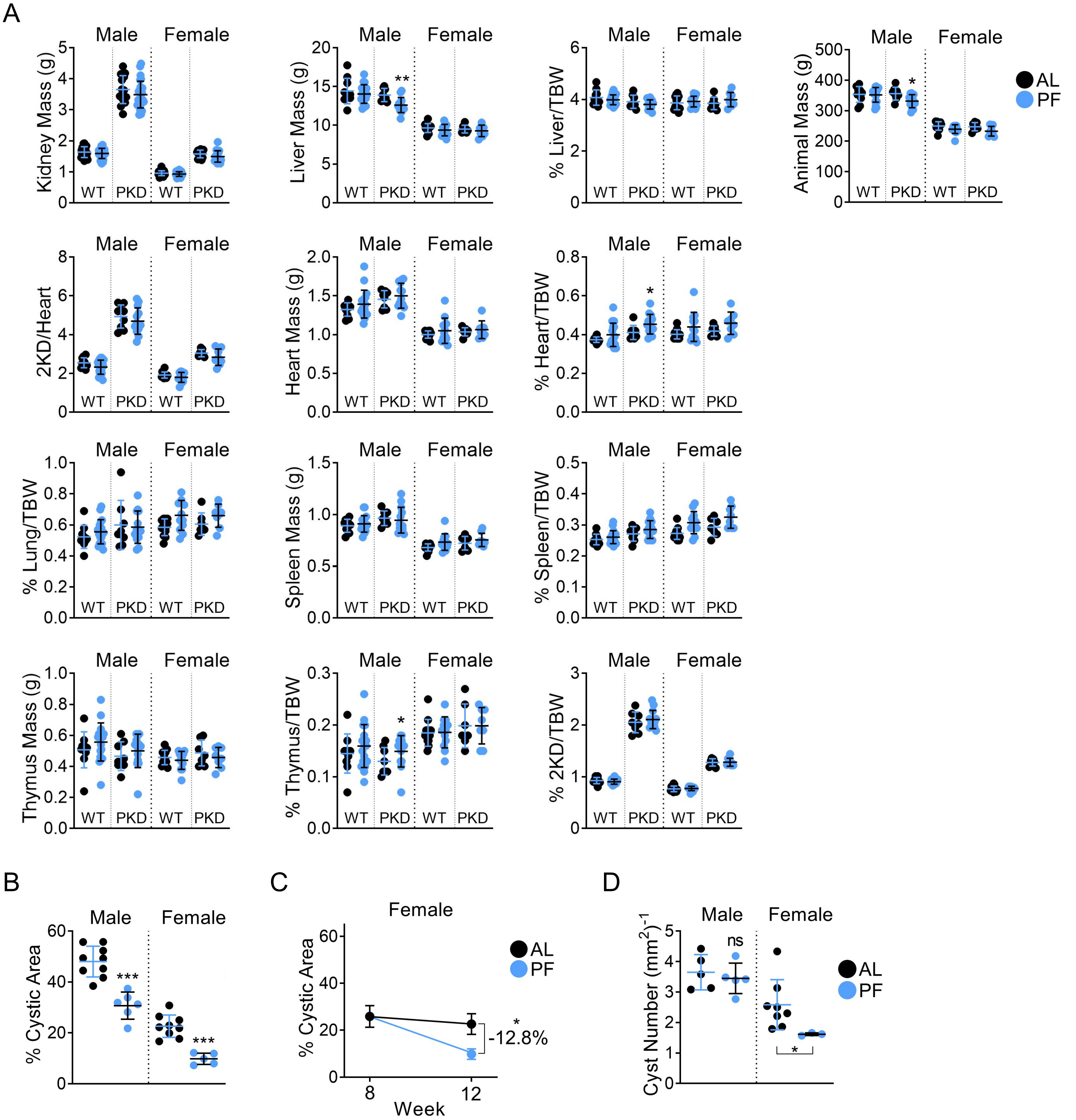

Supplemental Figure 4

Supplemental Figure 4: *Animal Measurements of Periodic Fasting Adult Male and Female Rats, Related to Figure 2 and Figure 3*

A) Tissue masses from 12-week-old ad libitum and periodic fasting male and female wild-type and polycystic rats. B) Animal masses of 12-week-old ad libitum and periodic fasting male and female wild-type and polycystic rats. B) Cystic area of ad libitum and periodic fasting male and female polycystic rats. C) Change in the cystic area between 8-week-old ad libitum and 12-week-old water and periodic fasting female polycystic rats. D) Cyst number per mm^2 of kidney from 12-week-old ad libitum and periodic fasting male and female polycystic rats.

(Mann-Whitney analysis was used to compare the mean between two groups. Mean and standard deviations are shown. *=P<0.05, **=P<0.01, ***=P<0.001)

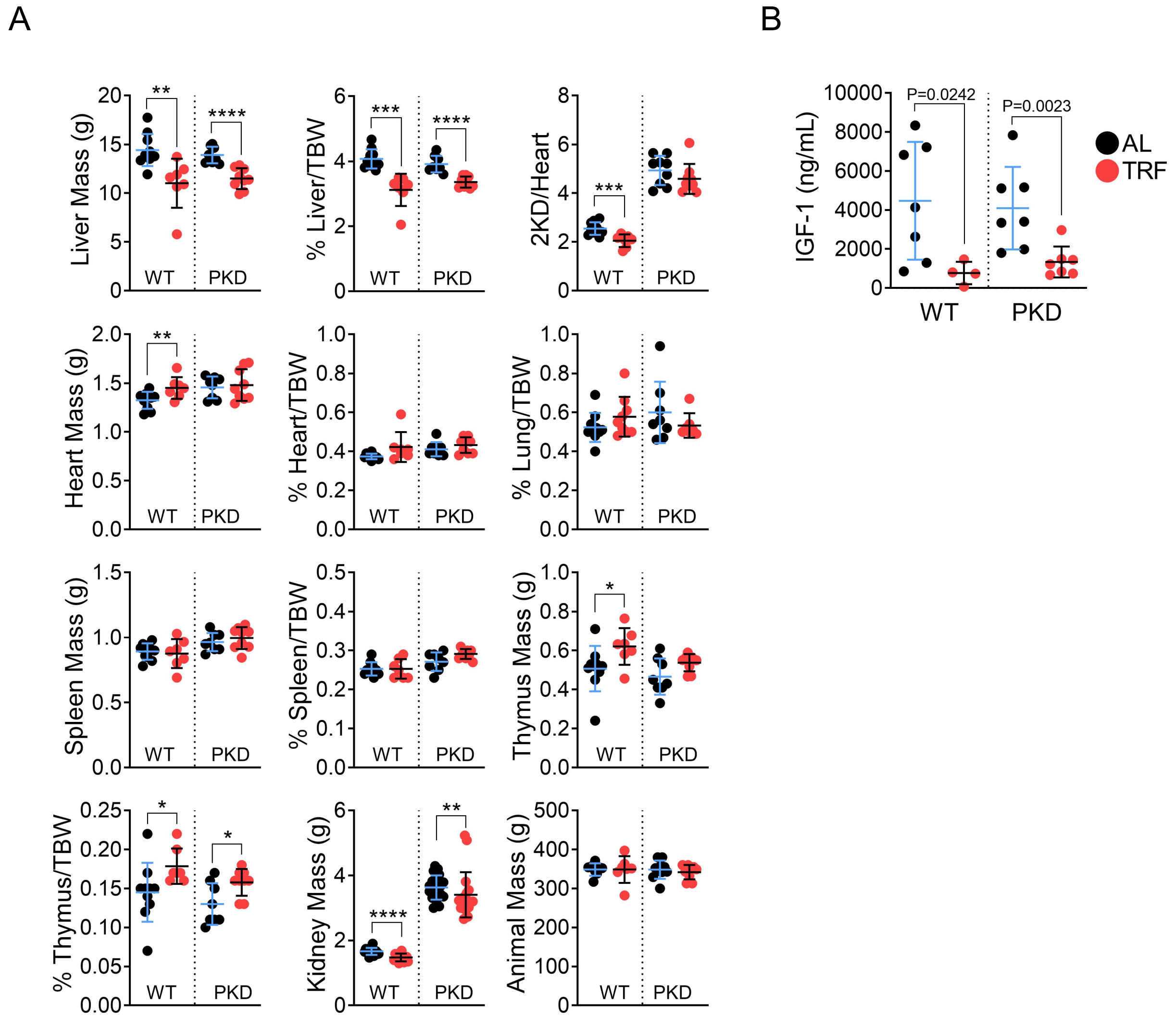

Supplemental Figure 5

Supplemental Figure 5: *Animal Measurements of Time-Restricted Adult Male and Female Rats, Related to Figure 2 and Figure 3*

A) Tissue masses of 12-week-old ad libitum and time-restricted male wild-type and polycystic rats. B) Serum IGF-1 levels from 12-week-old ad libitum and time-restricted feeding adult male wild-type and polycystic rats.

(Mann-Whitney analysis was used to compare the mean between two groups. Mean and standard deviations are shown. *=P<0.05, **=P<0.01, ***=P<0.001, ****=P<0.0001)

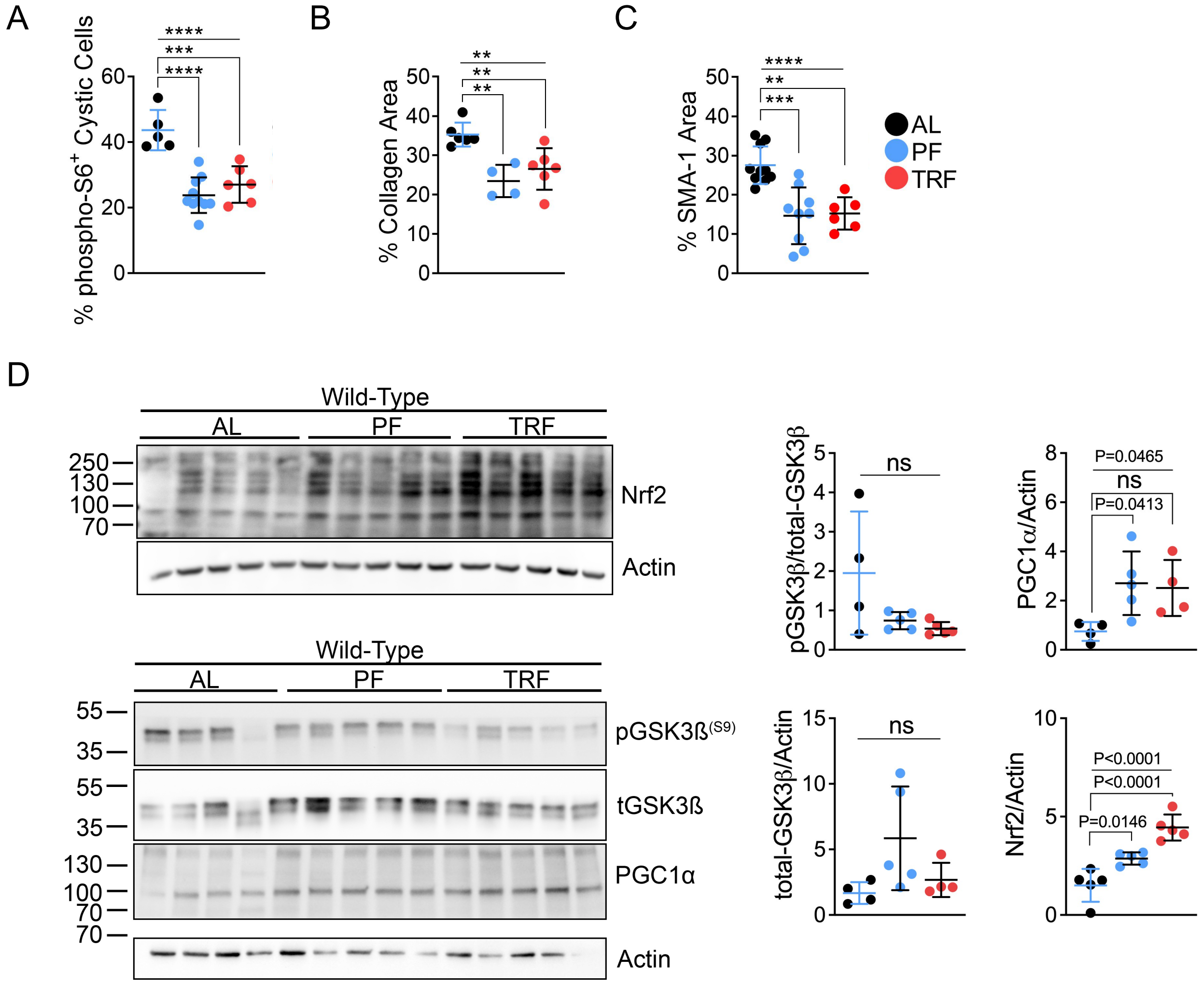

Supplemental Figure 6

Supplemental Figure 6: *Additional Measurements of Periodic Fasting and Time-Restricted Feeding Adult Male Rats, Related to Figure 2 and Figure 3*

A) Quantification of phospho-S6 cells within cystic epithelia from 12-week-old ad libitum, periodic fasting, and time-restricted male polycystic rats. B) Quantification of collagen in 12-week-old ad libitum, periodic fasting, and time-restricted male polycystic rats. C) Quantification of Smooth Muscle Actin (SMA-1) positive cells from 12-week-old ad libitum, periodic, and fasting time-restricted male polycystic rats. D) Western blot and quantification of 12-week-old ad libitum, periodic fasting, and time-restricted male wild-type rats.

(One-way ANOVA followed by ad hoc Tukey’s test was used for multiple comparisons. Mean and standard deviations are shown. **=P<0.01, ***=P<0.001, ****=P<0.0001)

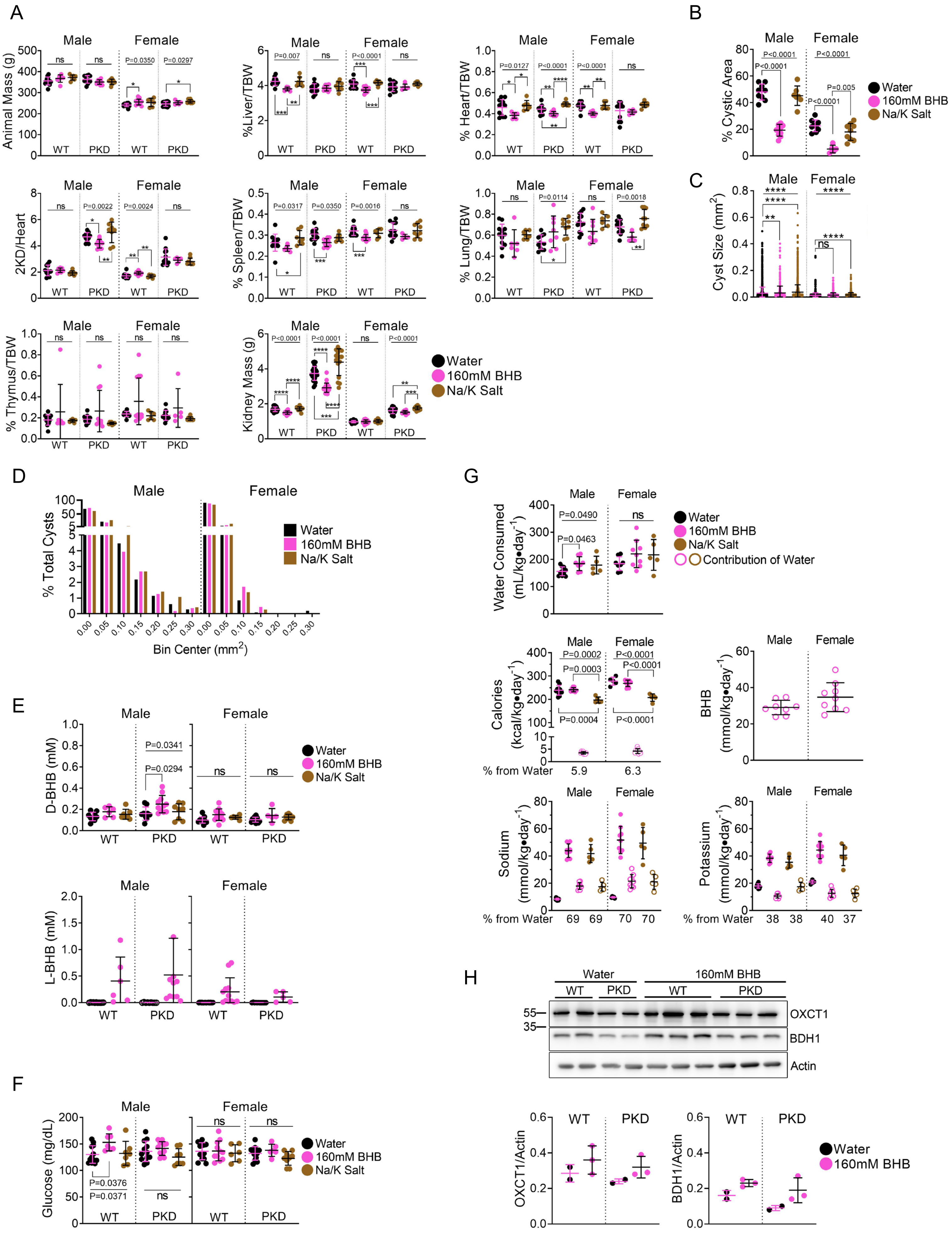

Supplemental Figure 7

Supplemental Figure 7: *Adult BHB-supplemented tissue masses and measurements* , Related to Figure 4 and Figure 5

A) Animal tissue masses from 12-week-old water, 160mM BHB, and salt-supplemented male and female wild-type and polycystic rats. B) Cystic area of 12-week-old water, 160mM BHB, and salt-supplemented male and female wild-type and polycystic rat kidneys. C) The size of cysts counted in Figure 4F from 12-week-old male and female polycystic rats. D) Percentage distribution of cysts by cyst size from 12-week-old water, 160mM BHB, and salt-supplemented male and female polycystic rats. E) Serum D-BHB and serum L-BHB levels from 12-week-old water, 160mM BHB, and salt-supplemented male and female wild-type and polycystic rats. F) Blood glucose values from 12-week-old water, 160mM BHB, and salt-supplemented male and female wild-type and polycystic rats. G) Food, water, BHB, sodium, and potassium consumption from water, 160mM BHB and salt-supplemented rats. Open circles depict the amount supplied by supplemented water versus that provided by food. H) Western blot and quantification of D-beta-hydroxybutyrate dehydrogenase (BDH1) and 3-oxoacid CoA-transferase 1 (OXCT1) from 12-week-old water and 160mM BHB male wild-type and polycystic rat kidneys.

(One-way ANOVA followed by ad hoc Tukey's test was used for multiple comparisons. Mean and standard deviations are shown. *=P<0.05, **=P<0.01, ***=P<0.001, ****=P<0.0001)

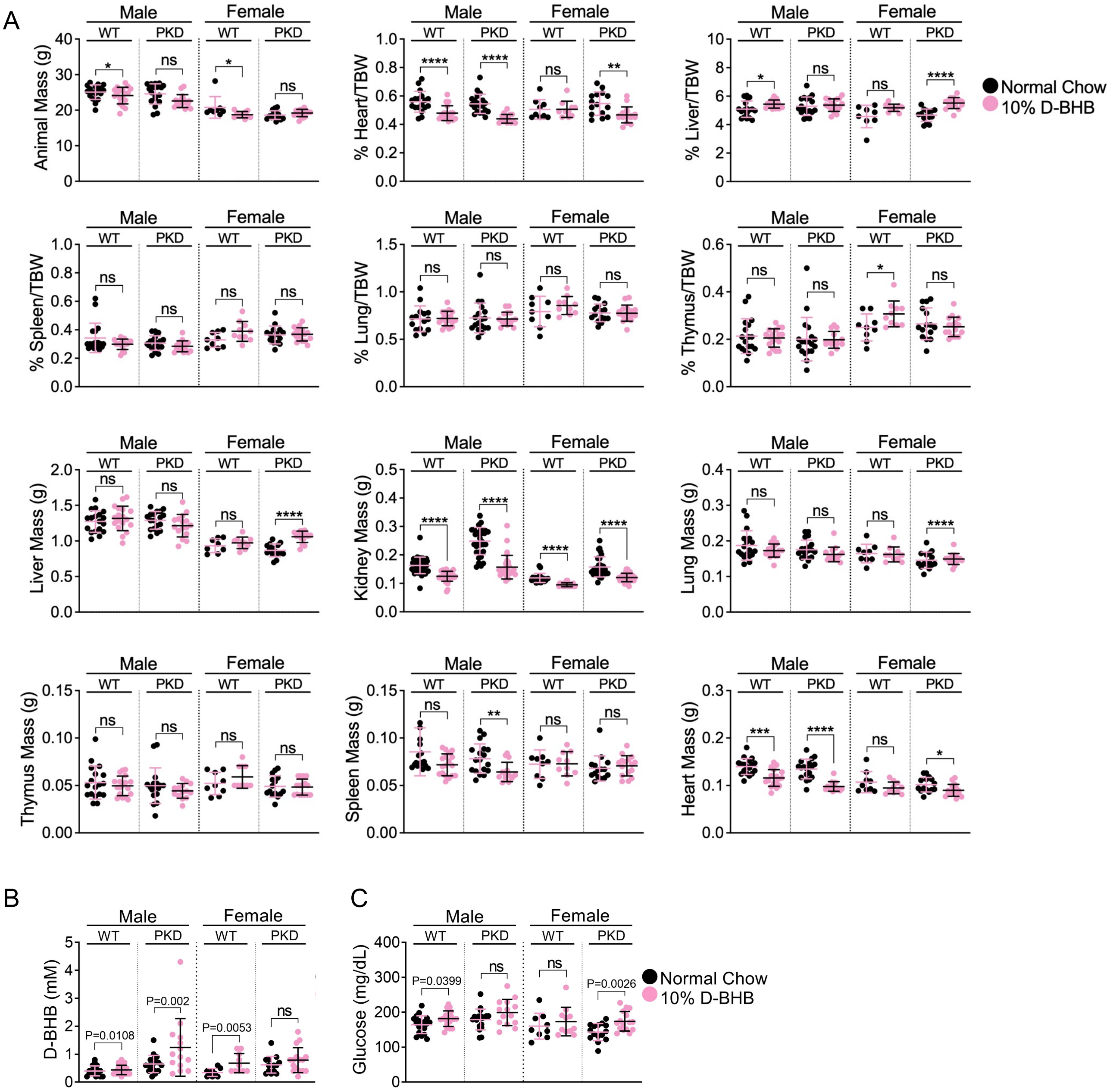

Supplemental Figure 8

Supplemental Figure 8: *Animal Measurements of 3-Month-Old Pkd1$^{+/+}$ and Pkd1$^{RC/RC}$ BHB-supplemented Mice, Related to Figure 6*

A) Tissue masses from 3-month-old male and female *Pkd1*$^{+/+}$ and *Pkd1*$^{RC/RC}$ mice. B) Blood D-BHB levels from 3-month-old male and female *Pkd1*$^{+/+}$ and *Pkd1*$^{RC/RC}$ mice. C) Blood Glucose levels from 3-month-old male and female *Pkd1*$^{+/+}$ and *Pkd1*$^{RC/RC}$ mice. (Mann-Whitney analysis was used to compare the mean between two groups. Mean and standard deviations are shown. *=P<0.05, **=P<0.01, ***=P<0.001, ****=P<0.0001)

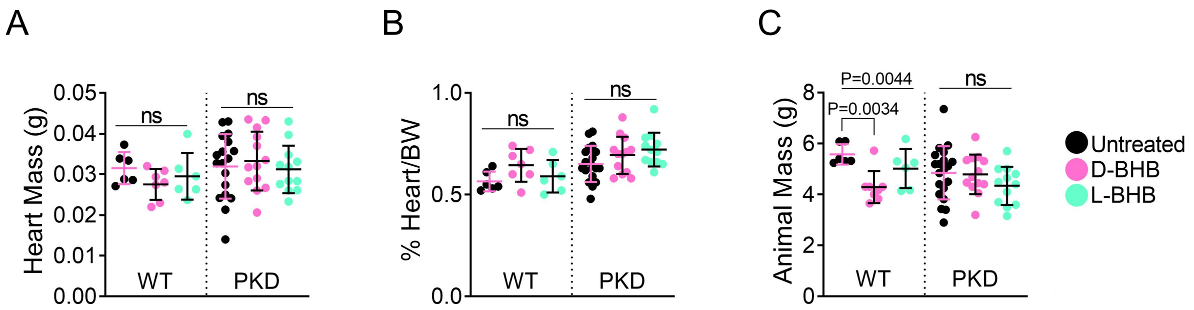

Supplemental Figure 9

Supplemental Figure 9: *Animal Measurements of Neonatal Pkd1-Ksp:Cre Mice Administered with Peritoneal BHB, Related to Figure 7*

A) Heart masses from P10 control, D-BHB-treated, and L-BHB-treated wild-type and *Pkd1*$^{fl/fl}$-Ksp:Cre mice. B) Heart over bodyweight ratio of P10 control, D-BHB-treated, or L-BHB-treated wild-type and *Pkd1*$^{fl/fl}$-Ksp:Cre mice. C) Animal masses from P10 control, D-BHB-treated, and L-BHB-treated wild-type and *Pkd1*$^{fl/fl}$-Ksp:Cre mice.

(One-way ANOVA followed by ad hoc Tukey's test was used for multiple comparisons. Mean and standard deviations are shown.)
